# Validated workflows for preparing and characterizing core-stained and surface-labeled fluorescent polymer particles with simple commercial automation tools

**DOI:** 10.1007/s00216-026-06443-z

**Published:** 2026-03-18

**Authors:** Abdelouahad El Abbassi, Paul Fürstenwerth, Christian Würth, Isabella Tavernaro, Ute Resch-Genger

**Affiliations:** 1 Division Biophotonics, Federal Institute for Materials Research and Testing (BAM), Richard-Willstätter-Str. 11, 12489 Berlin, Germany; 2 Department of Biology, Chemistry, and Pharmacy, Free University Berlin, Arnimallee 22, 14195 Berlin, Germany

**Keywords:** Automation, Fluorescent polymer beads, Nanomaterials, Dye staining, Fluorescent labeling, Validation

## Abstract

Preparation workflows of fluorescent nm- and µm-sized polymer particles used as reporters in fluorescent assays, bioimaging, and sensing studies or calibration tools for fluorescence methods in the life sciences can be time-consuming and labor-intensive. Also, the outcome can be operator dependent. Here, we present simple and cost-efficient automated workflows for dye loading and surface labeling of polystyrene particles (PSP), using a commercial self-programmable pipetting robot. For developing and fine-tuning automated staining workflows, hydrophobic Nile Red (NR) was incorporated into 100 nm, 200 nm, and 1000 nm PSP by an adapted swelling procedure of premanufactured particles in the presence of hydrophobic dyes, thereby confirming its reliability and versatility. Subsequent expansion of our automation concept to the labeling of carboxylated PSP with pH-sensitive 6-aminofluorescein (6-AMF) and aminated PSP with pH-responsive fluorescein isocyanate (FITC) demonstrated its broad applicability. All automated workflows were optimized and validated by gravimetry and spectroscopic measurements with a microtiter plate (MTP) reader in absorption and fluorescence mode to ensure particle recovery and reproducible fluorescence features and determine PSP dye loading and labeling efficiencies. Comparison with the manual fabrication of NR-stained PSP using an established swelling protocol showed that our automation approach utilizing a pipetting robot considerably reduced the variability in particle recovery and dye loading efficiency. Overall, our simple, labor- and time-efficient workflows with inexpensive and broadly available commercial automation tools present attractive alternatives to manual particle loading and labeling and provide the basis for fast parameter screening, parallel processing, and decreased hands-on time.

**Graphical Abstract:**

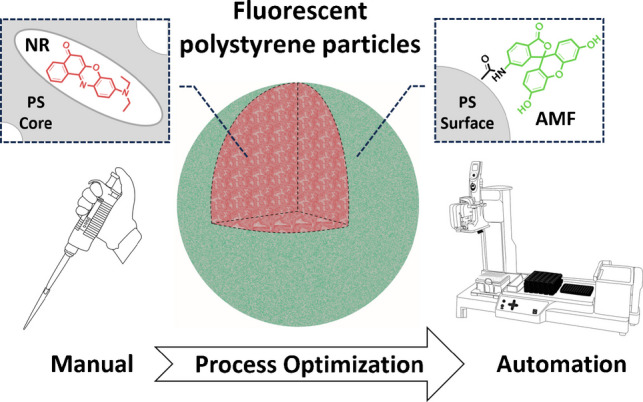

**Supplementary Information:**

The online version contains supplementary material available at 10.1007/s00216-026-06443-z.

## Introduction

Dye-loaded and labeled polymer micro- and nanoparticles are broadly utilized in the life and materials sciences [1–4]. Typical examples are bioanalytical assays, imaging studies, platforms for bead-based assays, and sensing, multiplexing, encoding, and calibration applications [5–7]. These particles are often made from chemically stable and biocompatible polymers such as polystyrene (PS) which are available from various manufacturers in different sizes with different surface functional groups. This enables the subsequent surface modification with recognition moieties, e.g., antibodies or DNA, antifouling agents such as polyethylene glycol (PEG) molecules or sensor dyes [7–13]. For non-crosslinked particles, staining with hydrophobic dyes such as Nile Red (NR) or sensor molecules can be realized by swelling with an organic solvent in the presence of the fluorophore [10, 14–16]. Drawbacks of this simple procedure, which can affect the quality of the resulting fluorescent beads, are the need to properly control the addition of organic solvents initiating bead swelling, reagent mixing, and incubation times as well as repeated washing and centrifugation steps to remove free and surface-adsorbed fluorophores [17]. The uncertainties introduced by these steps largely depend on the operator and can induce batch-to-batch variations in dye uptake and loading efficiency, bead fluorescence intensity and brightness, and particle concentration. Also, manual preparation steps are time-consuming and labor-intensive and can represent a bottleneck for large-scale or high-throughput synthesis of fluorescently stained or labeled particles. Automation which has meanwhile reached material chemistry and nanotechnology [18–23] can offer attractive solutions here. Examples for automation in nanotechnology present the automated syntheses of engineered nanoparticles (NP) including the screening of synthesis parameters to accelerate the identification of NP morphologies and chemical compositions providing optimum functionality, to improve the reproducibility of NP fabrication or to simplify frequent steps of NP preparation such as purification [18–23]. However, only very few literature reports exist on the automation of swelling-based dye loading or surface labeling for polymeric nanoparticles, despite its widespread use and scalability. In addition, the design, integration, and programming of devices for automating workflows are beyond the expertise of many researchers, and the tedious validation of such automated setups and so-called self-driven labs is very challenging.

**Fig. 1:**
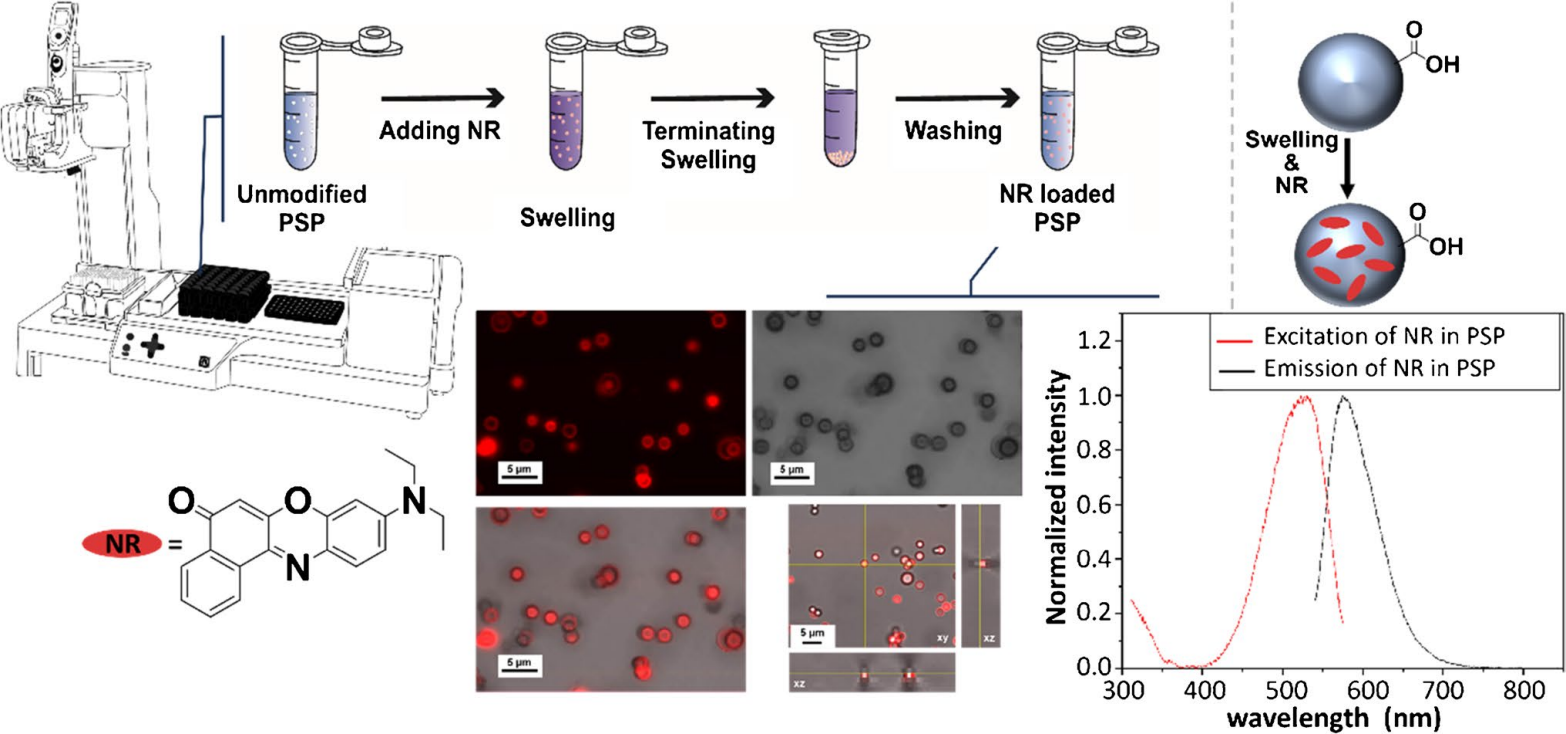
An overview of the workflow employed for the dye loading of polystyrene particles (PSP), indicating manual vs. automated steps and the optical characterization of the dye-stained PSP with optical spectroscopy and fluorescence microscopy. This workflow was representatively explored for PSP core staining with the hydrophobic neutral dye Nile Red (NR)

In this study, with the aim to develop strategies for automating workflows involved in the preparation, surface modification, and characterization of engineered nanomaterials and advanced materials such as particle-based fluorescent reporters and sensors with broadly available commercial automation tools, we utilize the effective one-step staining procedure of commercial PS particles (PSP) examined by Behnke et al. for many fluorophores from different dye classes [14, 15] to demonstrate the automation of the main workflows with a simple, self-programmable, commercial pipetting robot and a microtiter plate (MTP) reader (Fig. 1). Such self-programmable liquid handling systems have meanwhile been successfully applied in organic synthesis, bioconjugation, and nanoparticle formulation workflows, improving reproducibility, reducing operator effects, and increasing throughput [24–26]. With this broadly available device, we explored the automation of the liquid handling steps involved in the bead swelling and dye loading procedure, using PSP of different size and Nile Red (NR) as a model dye. Subsequently, this automation workflow was expanded to the labeling of common bead surface functionalities such as carboxylic acid and amino groups with reactive dyes such as pH-sensitive 6-aminofluorescein (6-AMF) or fluorescein isothiocyanate (FITC) previously employed by us for the fabrication of tricolored sensor beads and ratiometric sensor beads [10, 27], using different conjugation chemistries. These newly developed workflows involving the self-programmed pipetting robot reduce manual handling, allow parallel processing of multiple samples, and improve reproducibility and throughput while minimizing hands-on time and labor costs. Such simple and affordable approaches of integrating automation steps into the fabrication of functional particles using increasingly available commercial automation tools will pave the road to a more efficient and reproducible production of particle systems such as fluorescently stained and labeled polymer particles for research and clinical applications and ease workflow validation and standardization. This could also facilitate and accelerate, e.g., the production of particle-based reference materials, such as our spectral calibration beads developed for determining the wavelength dependent responsivity of imaging devices like fluorescence microscopes [16] and the fabrication of tailor-made reference particles for the life and environmental sciences for material-environment interaction, exposure and risk assessment studies [28, 29].

## Experimental

### Materials and methods

All chemicals, reagents, and solvents were of analytical grade or higher and used without further purification, unless specified otherwise. MilliQ water (0.055 μS/m, 18 Ω) was obtained from a Merck Milli-Q® IQ 700 system. Carboxylated polystyrene particles (PSP) with diameters of 100 nm (PPs-0.1COOH, Lot#: GK0006276842), 200 nm (PPs-0.2COOH, Lot#: GK0006530421), and 1 µm (PPs-1.0COOH, Lot#: GK1452201-01) and aminated polystyrene particles with a diameter of 1 µm (PPs-1.0NH2, Lot#: GK2092001-01) were purchased from Kisker Biotech. Nile Red (NR), tetrahydrofuran (THF), *N,N′*-dimethylformamide (DMF), methanol (MeOH), acetonitrile (ACN), 6-aminofluorescein (6-AMF), fluorescein isothiocyanate (FITC), and sodium *N*-hydroxysulfosuccinimid (s-NHS) were obtained from Sigma-Aldrich (Merck). *N*-(3-dimethylaminopropyl)-*N*′-ethylcarbodiimid-hydrochloride (EDC∙HCl) and 0.1 M sodium hydroxide solution were purchased from Carl Roth GmbH. Absolute ethanol (EtOH) for synthesis was purchased from Labsolute (Th. Geyer). Concentrated hydrochloric acid (HCl) was purchased from Chemsolute (Th. Geyer).

Automated pipetting was performed with an INTEGRA Bioscience ASSIST PLUS pipetting robot controlled by the VIALAB software (Version: 3.6.0, INTEGRA Biosciences), equipped with an INTEGRA Bioscience D-One (5–1250 µL) or an INTEGRA Biosciences Voyager 8-channel (1250 µL) pipette, as well as the Bioshaker 3000-T. The centrifugation steps of the manual and semi-automated approaches were performed with the centrifuge 5424 R from Eppendorf AG, while shaking and incubation were performed with a neoLabLine Rotator with the vortexer RM-2 M from neoLab and a sonication bath Palssonic PTIC-5-ES from ALLPAX. pH values were measured with a pH-meter SevenCompact™ S220 (Mettler Toledo) after a 3-point calibration at pH 4/7/10 using volumetric standards (Labsolute, Th.Geyer) under temperature correction. Absorption and fluorescence spectra were recorded with a Tecan Infinite M200 PRO microplate reader (MTP) using a 96-well quartz microplate (Hellma Analytics), and a FSP 920 spectrofluorometer from Edinburgh Instruments, using a 495 nm long-pass filter. Number-based hydrodynamic particle diameters (d_h,0_) and particle number concentrations (PNC) were assessed by nanoparticle tracking analysis (NTA) using a NanoSight LM 10 system from Malvern Panalytical equipped with a 405 nm laser. The measurements were performed at a temperature of 25 °C in static mode, following the standards ISO19430 and ASTM E2834 [30, 31]. The NanoSight NTA software (Version: 3.32) was used to capture 5 videos with 60 s and 25 fps of the scattering of the highly diluted samples. To ensure the reliability of the NTA results, the measurement sequence started with performance qualification (PQ) measurements using a dispersion of a particle size standard, nominally 107 nm polystyrene particles (PS-ST-B1261) from microparticles GmbH (SI, Figure S5). Dye loading and labeling of the micrometer-sized PS beads were qualitatively assessed by confocal laser scanning microscopy (CLSM), using an Olympus FV3000 equipped with a 100× oil objective.

### Manual PSP dye loading

100 nm PSPs were stained with NR using the swelling procedures previously reported by us using organic solvents such as THF and DMF and commercial premanufactured PSP surface-modified with carboxylic acid or amino groups [14]. Briefly, the dispersed PSPs were centrifuged at 21,130 rcf for 35 min and washed with MilliQ water to remove the storage buffer. Next, 24 mg (4.33∙10^13^ particles) of PSP was dispersed in 4.96 mL of MilliQ water and divided into eight 2 mL safe-lock centrifuge tubes from Eppendorf AG. To each tube, 200 µL of a NR solution (prepared in a 1:3 v/v mixture of THF:DMF with a concentration of 8.00 mmol/L) was rapidly added. The samples were incubated at room temperature (r.t., T = 23 °C) for 1 h under vigorous shaking, including four cycles of ultrasonication (2 min each). Subsequently, 0.50 mL of MilliQ water was added to stop the swelling process. The PSPs were centrifuged at 21,130 rcf for 35 min, washed once with ethanol and three times with MilliQ water, and finally redispersed in 1.32 mL of MilliQ water.

### Automated PSP dye loading

For the automated loading of the PSP with NR, a pipetting robot combined with a D-ONE pipette (5–1250 µL, INTEGRA Biosciences) with a manufacturer specified accuracy and precision for the working volumes, of about 2.5% and ≤ 0.7% for the 5–125 µL range, and 2.4% and ≤ 0.5% for the 50–1250 µL range, respectively, was employed to perform and optimize the key liquid handling steps, following a systematic optimization workflow (Figure S1). Initially, the robot was programmed to dispense 560 µL of MilliQ water into each of the 24 individual 2 mL safe-lock centrifuge tubes that were positioned on its deck. Subsequently, 60 µL of the PSP dispersion was added to each tube to achieve a final particle mass of 3 mg per sample, followed by automated mixing to ensure a uniform dispersion. Next, 200 µL of a NR solution of known dye concentration in a THF:DMF (1:3 v/v) mixture used to minimize THF evaporation was dispensed into each tube, using three different dye concentrations, i.e., (i) 2.00 mmol/L, (ii) 4.00 mmol/L, and (iii) 8.00 mmol/L, and eight replicate tubes per concentration. Then, the samples were transferred from the robot deck for the incubation step, consisting of four cycles of orbital shaking in a benchtop shaker, with each cycle being interrupted by 2 min of sonication in an ultrasonic bath to facilitate particle swelling and dye incorporation. These work steps were deliberately manually performed and not with the integrated shaking/heating module of the robot to allow for a comparison of solely the pipetting steps in the automated and manual PSP loading workflows. After the incubation step, the tubes were returned to the robot, and 500 µL of MilliQ water was added to each tube to terminate the swelling process. Centrifugation was done with a benchtop centrifuge at 21,130 rcf for 20 min for the 200 nm and 1000 nm PSP, and for 35 min for the 100 nm PSP, respectively. Next, the samples were returned to the robot platform, where 1 mL of the supernatant was removed, and the particles sequentially washed, first with ethanol, followed by three washing steps with MilliQ water. Finally, the PSPs were redispersed in MilliQ water (final volume of 1.32 mL) and manually sonicated to ensure homogeneous dispersion prior to further analysis.

### Absorption and fluorescence measurements with the dye-loaded and labeled PSPs

The optical characterization and the homogeneity studies of the particle dispersions were automated utilizing the pipetting robot to prepare samples with a particle concentration of 0.1 wt% in MilliQ water in a 96-microtiter plate using 200 µL of the particle dispersion per well. The microtiter plate was then transferred to an MTP reader for optical measurements. Thereby, extinction spectra were recorded from 300 to 800 nm and fluorescence emission spectra from 570 to 750 nm. Excitation was at the NR absorption maximum in the PSP of 550 nm. The extinction spectra were blank (baseline) corrected using MilliQ water filled wells as blanks. pH-dependent emission spectra have been measured in MilliQ water; the pH was adjusted with 0.1 M NaOH and 0.1 M HCl.

### Labeling of carboxylated PSP with pH-sensitive 6-AMF

Finally, we assessed the automated performance of a labeling protocol using the pipetting robot combined with the D-ONE (5–1250 µL, INTEGRA Biosciences) and D-ONE (0.5–300 µL, INTEGRA Biosciences, with a systematic error of 2.0% and a random error (precision) ≤ 0.60%) pipettes for 1 µm carboxylated NR-loaded PSP and the pH-sensitive dye 6-AMF bearing a reactive amine group [32]. Therefore, the NR-loaded 1-µm-sized beads (5 samples, 0.300 mg per tube) were each mixed with EDC∙HCl (0.025 mg, 0.16 µmol) and (0.035 mg, 0.16 µmol) s-NHS, both dissolved in 200 µL of MilliQ water adding up to 900 µL and stirred at r.t. for 10 min after each addition. An excess of 6-AMF (0.044 mg, 0.13 µmol) dissolved in 100 µL of MeOH was added to the particle dispersion. Further 500 µL of phosphate buffer saline (PBS) solution (pH 8.2) was added to the reaction mixture and the reaction mixture was shaken at r.t. for 2 h. The particles were centrifuged at 21,130 crf for 20 min and washed twice with MeOH and twice with MilliQ water. Finally, the particles were redispersed in 1.5 mL of MilliQ water.

### Labeling of aminated PSP with pH-sensitive FITC

Like the automated labeling of the carboxylated PS beads with 6-AMF, adapting a literature-known protocol [17], aminated NR-stained PSPs were labeled with FITC using the pipetting robot. The PSP dispersions were placed in 2 mL centrifuge units (3 mg/mL with phosphate buffer (0.1 M, pH 8.0), followed by addition of an excess of FITC in phosphate buffer containing 10 v% EtOH. The reaction mixtures were shaken for 3 h while being protected from light. The purification steps were the same as for the labeling procedure with 6-AMF, except that the first centrifugation/washing cycle was performed with a phosphate buffer containing 10 v% EtOH. More details are given in the Supporting Information (SI).

### Determination of particle loss

Gravimetric analysis of the obtained dye-loaded particles was performed in duplicate with a manual Cubis® MCM mass comparator 6.7 from Sartorius GmbH. To assess potential particle loss during the swelling procedure, aliquots of the final, well-sonicated PSP dispersion were pipetted into pre-dried and pre-weighed tubes, then dried again for at least 16 h at 80 °C and weighed to determine the residual particle mass. Thereby, the particle number concentration (PNC) was determined, a key factor for determining the loading efficiency. The results were validated by PNC measurements with NTA.

### Determination of dye loading efficiency and dye labeling

To determine the PSP dye loading efficiency, the NR-stained PSPs were dissolved in THF by mixing an aliquot of the aqueous particle sample with THF at a ratio of THF:MilliQ water of 4:1 (v/v). The mixture was shaken overnight (≥ 12 h) to ensure complete particle dissolution. Subsequently, the solution was transferred to three different wells of a 96-well plate, using 200 µL/well. The absorbance of each well was measured at 532 nm using an MTP reader. The dye concentration was then calculated using a calibration curve prepared from different dye concentrations in the same solvent mixture, covering a dye concentration range from 0.03 µM to 0.10 mM. The amount of 6-AMF bound to carboxylated PSP was determined in a similar way. The surface-labeled PSPs were dissolved in THF overnight (≥ 12 h), followed by evaporation of the solvent and redispersion in MeOH. The particle dispersion was incubated overnight (≥ 12 h). The resulting particle dispersion was then transferred to a 96-well microplate, using 200 µL/well. The absorbance at 480 nm of each well was measured. The dye amount was calculated from the aliquot used, employing a calibration curve covering the concentration range of 6–60 µM.

## Results and discussion

To cost-efficiently establish time-saving automation workflows in the preparation of fluorescent polymer particles using broadly available commercial automated laboratory devices, we first assessed automated workflows for our conventional swelling procedure of premanufactured non-crosslinked polymer particles for the fabrication of core-stained particle reporters and ratiometric particle sensors using the neutral dye NR [14, 15]. Thereby, we mainly focused on identifying and evaluating factors that influence the efficiency and reproducibility of the automated staining procedure compared to manual bead loading. As criteria to judge the applicability and reliability of this workflow, we used (i) the uniformity of bead staining, (ii) staining accuracy, (iii) PSP loading efficiency, and (iv) amount of bead loss or bead recovery rates. In addition, (v) we studied the potential of expanding this workflow to other application-relevant scenarios and higher throughput requirements and assessed the robustness of the adapted workflows. Furthermore, (vi) we explored the implications of these findings on up-scalability and potential integration into automated platforms, highlighting both the advantages and limitations observed during the implementation of the different work steps.

**Fig. 2:**
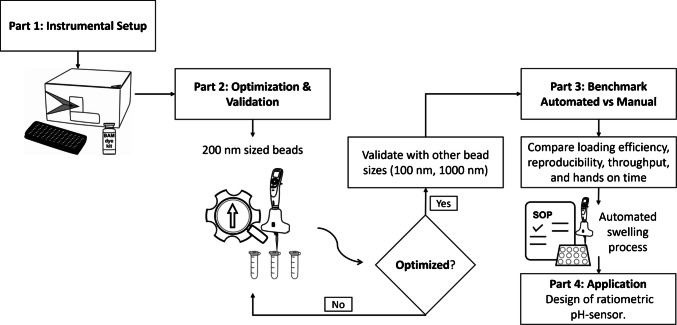
Workflows of the automated loading of the carboxylated PSP with the exemplarily chosen solvatochromic dye Nile Red (NR) and labeling of carboxylic acid surface groups with the pH-sensitive dye 6-aminofluorescein (6-AMF) or surface amine groups with the pH-sensitive dye fluorescein isothiocyanate (FITC) using amide coupling chemistry. Part 1 of the workflow includes the evaluation of the filling of the microtiter plate with the pipetting robot and the readout of the microtiter plate reader (MTP) with a test dye (the BAM spectral fluorescence standard dye Y) that was validated together with the BAM fluorescent calibration kit dyes BAM-F001 to BAM-F005 [33]. In workflow Part 2, the swelling protocol was assessed and optimized using 200 nm PSPs and three concentrations of NR, including the tuning of the robot parameters aspiration/dispense height, speed, and solvent mixing. Additionally, dye loading efficiency, particle recovery, and reproducibility were determined

The final workflow using a common pipetting robot, a centrifuge, a vortex mixer, and a MTP is presented in Fig. 2*.* As shown in this figure, the workflow consists of four steps. (1) Performance validation of the filling of the microtiter plate with the pipetting robot and the performance of the optical readout with the MTP, using the test dye Y, previously validated together with the BAM certified reference dyes BAM-F001 to BAM-F005 from the BAM-FCalKit [33, 34]. (2) Optimizing the automated workflow of PSP dye loading by particle swelling and deswelling cycles for 200 nm PSP and three NR concentrations by adjusting the pipetting robot parameters aspiration and dispense height and speed, followed by assessing the versatility of the optimized staining protocol by NR loading of 100 nm and 1000 nm PSP, and (3) comparing automated and manual swelling workflows for optimized dye staining conditions, exploiting dye loading efficiency, reproducibility, throughput, and hands-on time as evaluation parameters. Thereby, a versatile standard operation procedure (SOP) including instrument settings and acceptance criteria was derived. (4) Finally, this optimized automated approach was expanded to the preparation of ratiometric pH sensor particles by integration of a labeling step of common surface functional groups on PSP such as carboxylic acid and amine groups using reactive dyes and different conjugation chemistries.

### Assessing the optical readout workflow and MTP reader performance

**Fig. 3:**
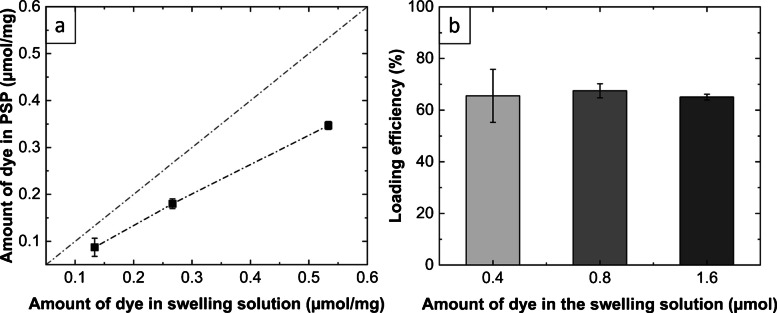
Loading of 200 nm PSP with the dye NR as a function of the initial amount of the dye in the swelling solution. **a** Content of NR incorporated into PSP as a function of the dye amount used during bead swelling. The data represents the mean ± SD of three independent runs with n = 8 samples per run measured. The dashed line represents a dye incorporation efficiency of 100%. **b** NR loading efficiencies calculated from these data sets

To assess the reliable loading of the microtiter plates (MTP) with the pipetting robot and the reliable readout of the solutions filled into transparent quartz MTP (absorbance measurements) and black quartz MTP (fluorescence measurements, to avoid crosstalk) with the MTP reader, we performed control experiments with two dye solutions. The quartz MTP shown in the SI in Figure S2a) were employed because the use of organic solvents such as THF is not compatible with conventional MTP made from polymers such as PS. Possible sources of uncertainty assessed prior to our particle loading and labeling studies included the evaporation of volatile organic solvents for these measurements during MTP scanning, changing the filling height and meniscus of the measured solutions over time and the homogeneity of MTP illumination which can affect the intensity of measured fluorescence spectra [35, 36]. These control measurements were performed with an acetonitrile (ACN) solution of the BAM standard dye Y, previously assessed in an interlaboratory comparison on spectral correction of fluorescence spectra [34]. ACN has only about half the vapor pressure of THF at 20 °C. It was used for the PSP swelling procedure and the dissolution of dye-loaded and/or labeled PSP to determine these properties in solution without distorting the scattering of the dispersions induced by the presence of the PSP [15, 32]. Absorption measurements with a 0.001 mM solution of NR in THF:MilliQ water (4:1; v:v) were also performed (see SI, Fig. 3b). Absorbance measurements with ACN solutions of dye Y revealed relatively small well-to-well variations in the absorbance data, and minor changes in absorbance intensity (see SI Figure S2b and Figure S3c). For fluorescence measurements, monitoring dye fluorescence intensity at the respective emission maxima over time, a more pronounced gradual decrease in emission intensity was observed tentatively attributed to evaporation-induced changes in well filling height and meniscus. For hydrophobic and solvatochromic NR dissolved in a THF:MilliQ water mixture, also an evaporation-induced increase in water content over time can lead to a reduction in fluorescence quantum yield and hence measured fluorescence intensity. Moreover, this could favor the adsorption of the hydrophobic dye onto the MTP well surfaces due to the accordingly reduced dye solubility. To minimize solvent evaporation during the automated swelling procedure and during pipetting and incubation steps, subsequently, we employed the solvent system DMF:THF of 3:1 (v/v), equally suited for PSP swelling. For the determination of the dye loading and labeling concentration of the beads, a mixture of THF:MilliQ water of 4:1 (v/v) was employed as these measurements were performed on a much faster time scale.

### Optimization of the automated swelling procedure

Next, we optimized the automated PSP swelling protocol first for 200 nm PSP. The reproducibility of the swelling procedure was assessed by preparing eight samples per dye concentration, each in a separate centrifugation tube (SI, Figure S4). Special emphasis was dedicated to the automated pipetting steps without considering, e.g., a possible influence of the shaking unit. Please note that shaking can also be performed by the robot, using the Bioshaker 3000-T unit to further reduce hands-on time, but this step was not considered in the comparison of automated and manual workflows.

**Table 1:** Gravimetric determination of the average mass of 200 nm PSP after the automated swelling routine: values reported as mean ± standard deviation (SD) derived from eight replicates per dye loading concentration (mM) for three runs of the automated loading workflow using dye loading concentrations of 2, 4, and 8 mM. respectively. The PSP starting concentration was 3 mg/replicate. The calculated PNCs were compared to NTA measurements (for more details see SI)

Dye loading (mM)	Run 1 (mg)	Run 2 (mg)	Run 3 (mg)
2.00	1.7 ± 0.7	2.6 ± 0.2	2.5 ± 0.2
4.00	1.7 ± 0.4	2.2 ± 0.1	2.2 ± 0.1
8.00	1.6 ± 0.5	2.2 ± 0.1	2.2 ± 0.1

To optimize and validate the automated pipetting routines, three dye concentrations, i.e., (i) 2.00 mmol/L, (ii) 4.00 mmol/L, and (iii) 8.00 mmol/L, were assessed, each for eight replicates in three independent runs. Particle recovery rates summarized in Table 1 were gravimetrically determined and validated via NTA measurements (for more details, see SI, Figure S6). The lower average recovery values observed during the initial optimization run (Run 1) of 55% compared to the subsequent validation runs (Runs 2 and 3) yielding recovery rates of 77% originate from non-optimized pipetting parameters in Run 1, particularly the immersion depth of the robotic tip and the dispensing speed during washing steps. Refinement of these parameters increased the average particle recovery and reduced the variability among replicates as shown in Table 1. The good reproducibility of the gravimetrically determined values confirms the reliability and robustness of the optimized automated protocol for the swelling workflow once handling parameters have been optimized. All subsequent measurements and data were collected with optimized pipetting parameters.

To determine the dye loading efficiency, we examined the influence of the dye concentration in the swelling solution on the amount of PSP incorporated dye, measuring three independent replicates of the loading procedure for each dye concentration (see the “Experimental” section), each comprising eight centrifuge tubes. The results are shown in Fig. 3a. The amount of PSP incorporated dye consistently increased with increasing initial dye concentration. The relative standard deviations decreased from about 13% for the lowest dye concentration to 8% for the intermediate and 1% for the highest NR concentration. This demonstrates the excellent reproducibility of the automated swelling procedure. The concentration-dependent increase in the concentration of incorporated dye, combined with the low variability across independently prepared samples, confirms the robustness of the automated method and its suitability for controlled dye incorporation into PSP. Calculation of the dye loading efficiency of the 200 nm PSP per mg of PSP particles provided values of 65% ± 10%, 67% ± 3%, and 65% ± 1% for the low, intermediate, and high initial dye concentrations, respectively (see Fig. 3b). This suggests that for the swelling solvent used, i.e., DMF:THF = 3:1 (v:v), the dye uptake efficiency remained stable, pointing to a loading limit for a given particle size and swelling solvent composition. Subsequently, the automated swelling protocol optimized for 200 nm PSP was applied to other PSP of smaller and larger sizes, i.e., to 100 nm and 1000 nm PSP to assess its robustness and scalability. Gravimetric analysis of bead recovery after swelling revealed a considerable influence of particle size on the recovery efficiency (see also SI, Table S2, for more details). While both 200 nm and 1000 nm PSP showed comparable recovery rates of 78%, with low variability for the replicates, for 100 nm PSP, lower and more variable recovery rates of 49% were observed. This is ascribed to the efficient formation of pellets during centrifugation for 200 nm and 1000 nm PSP that can be hence washed with minimum particle loss. In contrast, 100 nm PSP do not form stable pellets during centrifugation, making them more susceptible to particle loss during washing and redispersion steps, induced apparently already by small disturbances caused by the pipetting steps, reducing the number of recovered particles (Table S1). The determination of the hydrodynamic radii by NTA and comparison to those of the initially applied particles (SI, Figure S7) demonstrated that the particle size of 100 nm and 200 nm PSP is not affected by the swelling procedure; in addition, the particle size distributions are not significantly affected. Overall, these results confirm that the optimized swelling protocol is broadly applicable to differently sized PSP. For the more challenging loading of PSP with sizes < 100 nm, apparently further optimization is needed for improved recovery rates and reduced bead loss.

**Fig. 4:**
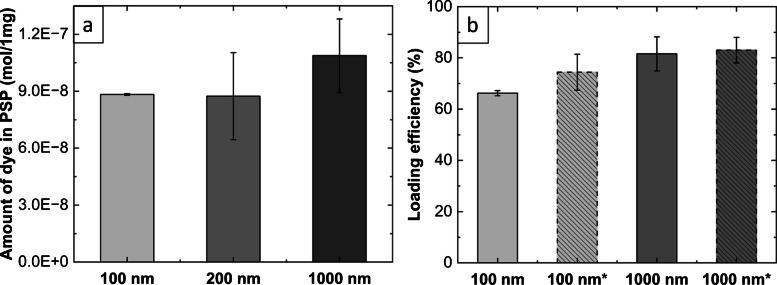
Validation of the automated swelling workflow for different PSP sizes. **a** Amount of incorporated NR as a function of particle diameter for identical swelling conditions (initial dye amount: 0.4 µmol). **b** Corresponding dye loading efficiency of the differently sized PSPs. The bars represent mean ± SD values derived from three independent runs with n = 8 samples per run. The values marked with (*) denote data taken from previous measurements of Laux et al. [32] included for direct comparison with the automated method.

As follows from Fig. 4a illustrating the influence of particle size on the average amount of incorporated NR obtained for similar swelling and loading conditions, values of about 0.88 µmol/mg for 100 nm, 0.87 µmol/mg for 200 nm, and 1.09 µmol/mg for 1000 nm PSP were obtained. The similar values obtained for the 100 nm and 200 nm particles indicate that, when normalized to the particle mass, dye incorporation is largely independent of particle size in this size regime. In contrast, the 1000 nm PSP exhibits a higher mean loading degree, which is, however, associated with a significantly larger SD value, likely due to the rapid sedimentation of the larger beads during the loading process. This sedimentation reduces the homogeneity of the swelling environment and can introduce variation amongst replicate tubes. In contrast, the 100 nm and 200 nm PSPs remain well dispersed during processing and exhibit significantly lower variabilities. The loading efficiencies shown in Fig. 4b indicate a similar trend. The 100 nm PSP revealed a loading efficiency of about 66% comparable to the value observed for the 200 nm PSP. To assess whether surface adsorption contributes to this observation, Zeta potential measurements were performed before and after the dye loading procedure of the PSP (SI, Figure S8). The negligible change in surface charge supports the absence of dye adsorption on the PSP surface during the PSP loading process. Overall, the loading efficiencies determined from the automated method closely match the data reported by Laux et al. [32] for 100 nm and 1000 nm PSPs determined for manual loading. This supports the reliability and validity of our automated swelling workflow.

Taken together, the results highlighted in Fig. 4 demonstrate that our automated loading workflow optimized for 200 nm beads can be easily translated to both smaller and larger particles. With the automated swelling workflow, dyes can be reliably and reproducibly incorporated into PSP for a wide particle size range with size-related sedimentation effects slightly reducing particle loading homogeneity and reproducibility. Although larger beads can allow for higher loading efficiencies, their faster sedimentation requires an adjustment of the handling procedure to minimize increased tube-to-tube variation. Using our automated swelling procedure, up to 24–48 centrifuge tubes, each containing 3 mg of PSP, can be processed in a single run with either varying dye concentrations for a given particle size or for different particle sizes for a constant dye concentration, highlighting the potential of this simple workflow for higher throughput applications. Please note that for the inexpensive instrumentation employed in this proof-of-concept study, the total number of tubes is limited by the vacant positions on the pipetting robot and the centrifuge. Hence, with slightly more advanced equipment, throughput can be easily increased.

### Automated vs. manual loading workflow—influence of the operator

Next, we compared automated and manually performed particle loading exemplarily for 100 nm PSP to assess the reproducibility and the advantages and limitations of each workflow. This comparison included operators performing the procedure for the first time and well-trained operators who had performed the dye loading of particles via this swelling procedure before. For manual pipetting, different sets of Eppendorf Research Plus single-channel variable pipettes were used, covering volume ranges of 20–200 μL (yellow), 30–300 μL (orange), and 100–1000 μL (blue), along with the recommended epT.I.P.S. (Eppendorf AG). All operators could choose from calibrated pipettes, all having a systematic volume error of < 4%. While automated bead loading was completed within 30 min, all manual pipetting steps were completed within 1.5–2.5 h, even for well-trained staff. This highlights the reduced hands-on time enabled by our automated workflow.

**Fig. 5:**
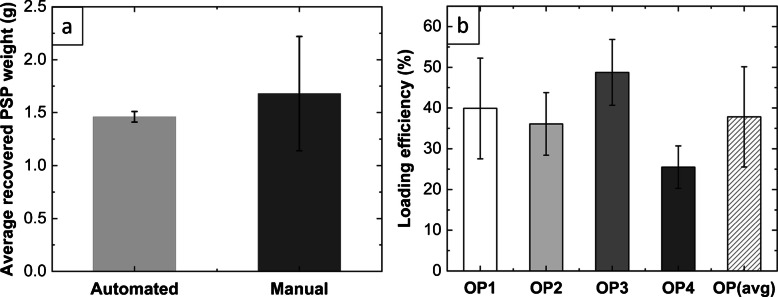
**a** Comparison of particle recovery for the automated and manually performed PSP swelling and NR loading procedures. **b** Comparison of the average loading efficiency for the manual swelling procedure performed by different operators of varying levels of expertise and training. The additional bar represents the overall average loading efficiency ± SD across all operators

Figure 5a shows a comparison of the particle recovery rates obtained by the automated workflow and manual dye PSP staining, performed by four operators. The automated process enabled a consistent recovery of 1.46 ± 0.05 mg with excellent reproducibility for three independent runs. The manual procedure yielded a higher average recovery rate (1.68 ± 0.54 mg), however, accompanied by significantly larger SD values for the four operators. The significant variations in particle recovery rate between the four operators demonstrate the considerable influence of uncertainties related to operator performance of pipetting, mixing, and washing steps for the manual workflow which all contribute to particle loss during handling. This trend is also reflected by the loading efficiencies obtained for the manual workflow shown in Fig. 5b, which vary by 25–49% depending on the operator. This equals a variability by almost a factor of two between the best and poorest operator performance. The relatively large SD values support the lower reproducibility associated with manual handling. For the automated workflow, due to better process control, variabilities originating from differences in pipetting accuracy, bead resuspension efficiency, timing during the swelling steps, and bead loss during washing or tube transfers factors can be eliminated. This comparison clearly highlights the advantages of workflow automation, enabling a reduction in the coefficient of variation by a factor of about 10, i.e., from 32% for the manual workflow to 3.4% for the automated PSP loading procedure (see also Fig. 5a).

### PSP labeling with pH-sensitive 6-AMF and FITC

Subsequently, we integrated a labeling step into our automated workflow for the fabrication of fluorescent particles for common surface functionalities at bead surfaces and frequently utilized conjugation chemistries. Thereby, we aimed for the preparation of ratiometric particle sensors in a single workflow, first preparing core-stained PSP using an analyte non-responsive or inert reference dye, here NR, and subsequently covalently attaching a reactive, water-soluble analyte-responsive dye to surface functionalities at the bead surface. Surface labeling ensures sensor dye exposure to the aqueous environment and accessibility to analytes in the aqueous phase. This workflow can be applied to all types of surface functionalized particles, i.e., organic polymer and inorganic particles. For the development of the first proof-of-concept workflow, we chose carboxylated PSP and the pH-responsive dye 6-AMF. NR and 6-AMF are excitable at the same wavelength and reveal spectrally distinguishable emission spectra. Single wavelength excitation of the reference and analyte-responsive dyes is an important prerequisite for the design of ratiometric sensors, thereby compensating for fluctuations in the excitation light intensity which could otherwise differently affect the fluorescence intensities of both dyes, while spectral discrimination between the fluorescence of the reference and sensor dyes is required for accurate signal differentiation and signal referencing by reading out signal quotients. The combination of pH-inert NR and pH-responsive 6-AMF, which was successfully used by us before for the design of ratiometric pH sensors, e.g., for pH measurements in biofilms [27], allows for ratiometric pH sensing in the pH range of about 6–9 and at more basic pH values.

**Fig. 6:**
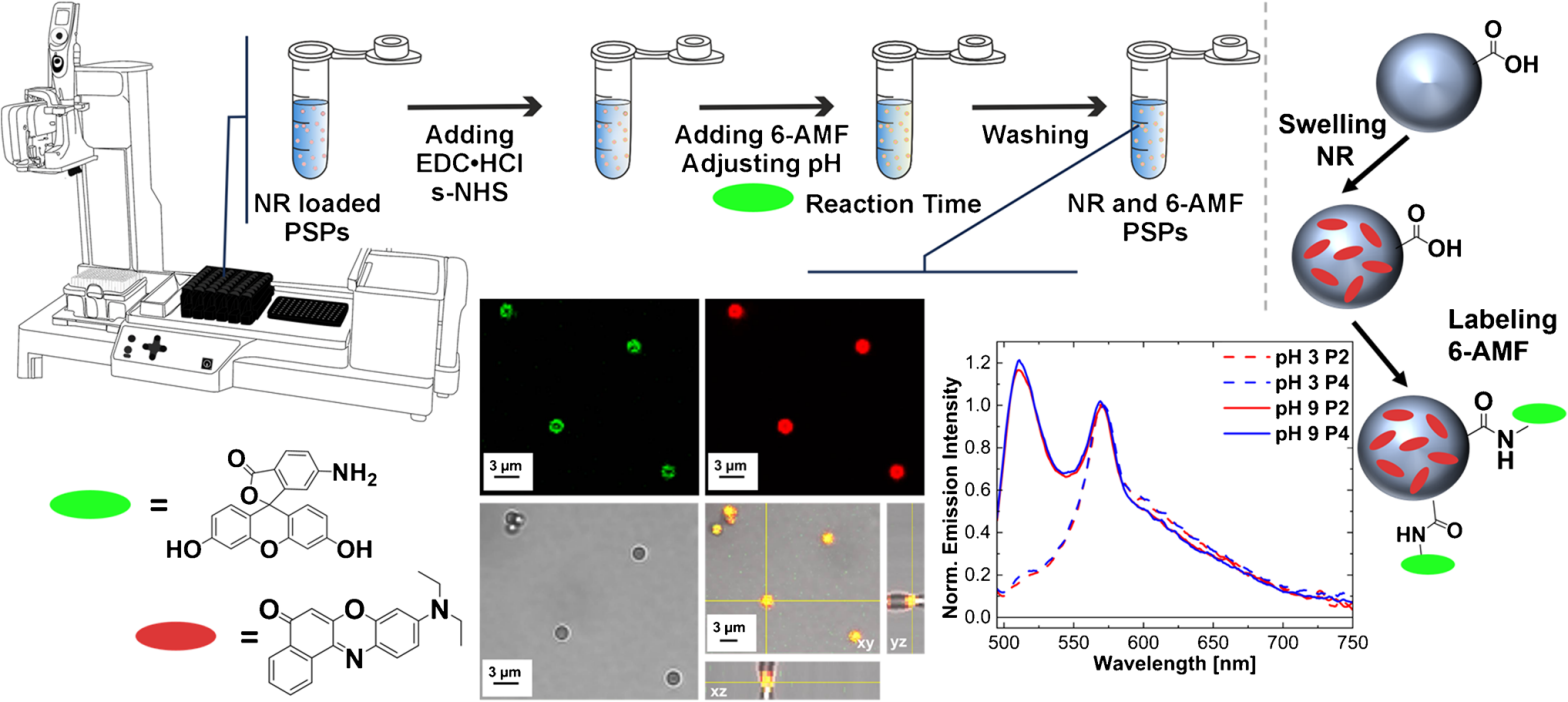
Left: Overview of the work steps involved in the automated labeling workflow of carboxylated PSP (COOH-PSP) with 6-AMF, thereby covalently binding 6-AMF onto the PSP surface. In the bottom, the normalized emission spectra and fluorescence intensity of the ratiometric sensor system at pH values of 3 and 9 and microscopic images of the NR-loaded and 6-AMF-labeled particles are shown, revealing the 6-AMF (green, Filter 500–540 nm; visible at a pH of about and increasing at more basic pH values) and NR (red, Filter 570–670 nm; always ON) emission upon excitation at 488 nm. The emission spectra of the dyes shown at pH values of 3 and 9 were recorded with a 495 nm long-pass filter; fluorescence excitation was at 480 nm. Right: Schematic procedure for the NR loading and 6-AMF labeling of COOH-PSP

As shown in Fig. 6, excitation at 480 nm leads to the fluorescence of NR in the PSP core and 6-AMF attached to the PSP surface, revealing emission maxima at 512 nm and 575 nm, respectively. The 6-AMF fluorescence is, however, only visible for neutral and basic pH values, while the fluorescence of NR is pH-independent. The CLSM images, recorded upon excitation at 488 nm, show the emission of NR (red) and 6-AMF (green) (see Fig. 6 and SI, Figure S10) of five sensor particle batches produced by our automated labeling workflow. Strong luminescence was visible for both the NR-stained (see *SI*, Figure S9) and the 6-AMF-labeled and NR core-stained PSP batches (Figure S10). The mean 6-AMF labeling concentration was optically determined to be 31 ± 4 nmol, using a similar photometric method, as described in the “Experimental” section and in the SI in Figures S11a and b, with the observed small SD values derived from measurements of these 5 bead batches underlining the good reproducibility of our automated labeling workflow. Next, we explored an alternative conjugation chemistry commonly employed using aminated PSPs and the pH-sensitive dye fluorescein isothiocyanate (FITC) to demonstrate the versatility of our labeling workflow. Also in this case, core staining with varying concentrations of NR and labeling with different amounts of pH-sensitive FITC could be successfully achieved using our simple automated labeling workflow and allowed for the fine-tuning of the particle performance (SI, Figure S12).

These labeling studies reveal that our simple automation concept can be used for single-step particle loading or labeling processes and for two-step protocols for sequential particle loading and labeling, with a minimum number of still manually performed work steps. Thereby, the simultaneous preparation of multiple batches of core-stained and/or surface-labeled fluorescent particles using different parameters such as dye loading or labeling concentration and solvent mixture is feasible for different particle sizes and conjugation chemistries without the need for protocol adjustment that would complicate manual performance, and surface-labeled particles.

### Time consumption and workflow efficiency

**Fig. 7:**
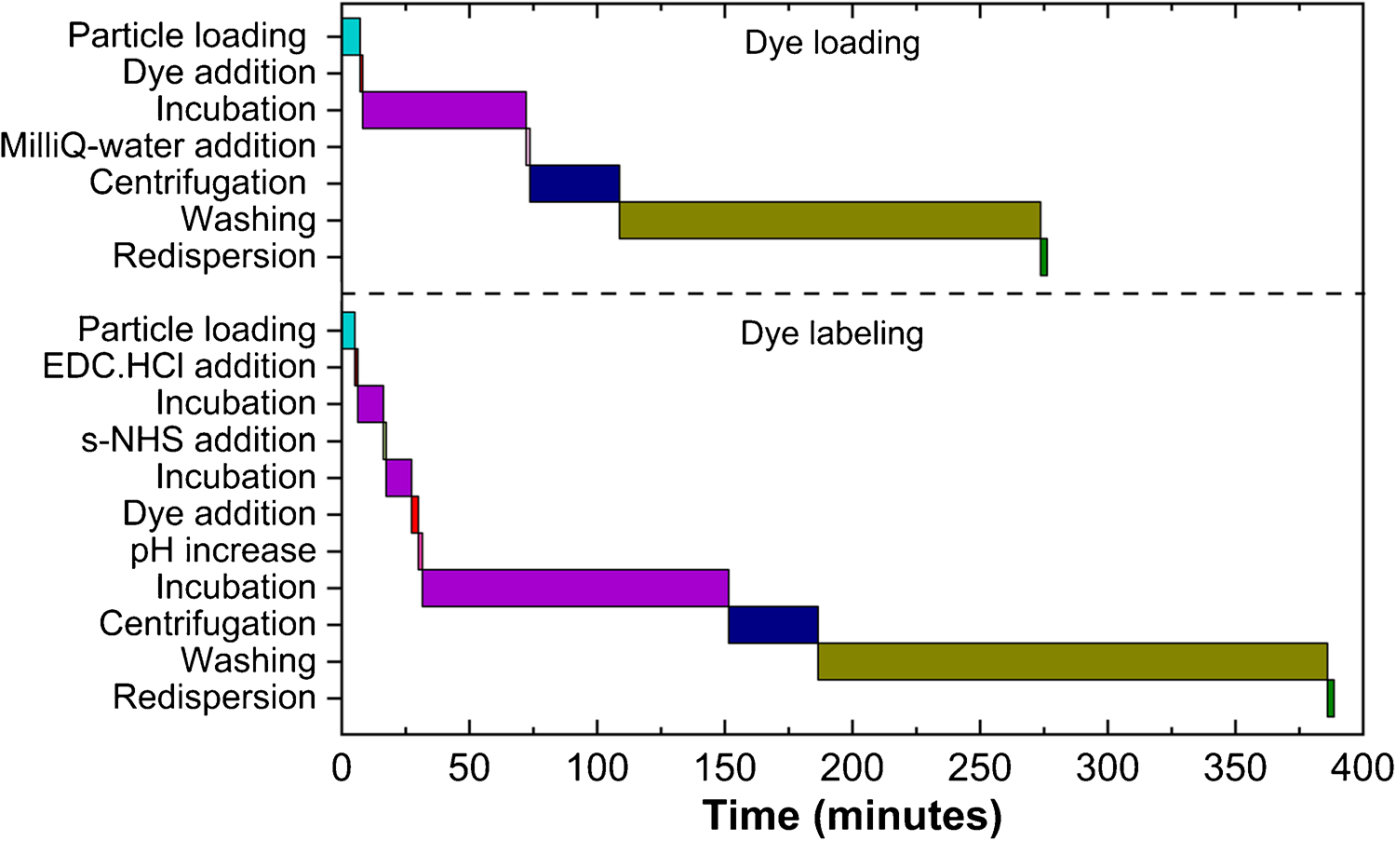
Overview of the process duration and time required for each work step of the automated dye loading and surface labeling of PSP

Finally, we assessed the reduction in hands-on time, overall time savings, and workflow efficiency achieved by the automated workflow for the dye loading and surface labeling of PSP. As the in-line characterization of the optical properties of the resulting fluorescent PSP was always performed with an MTP reader that complies with automated characterization workflows, this work step was not considered. As shown in Fig. 7, work step automation increased the process efficiency compared to manual handling, as the hands-on time could be reduced from several hours to less than 10–15 min, limiting manual work steps only to the necessary manual tube transfer to and from the shaker and centrifuge, while the robot autonomously performed all fluid-handling tasks. For manual bead staining, the total time for dye loading was about 4 h 36 min at maximum, whereas the pipetting robot equipped with the Bioshaker 3000-T completed the pipetting process in about 109 min, including the usage of 49 tips, four centrifugation runs (20–35 min each), and minimal remaining hands-on time. For particle labeling, the total estimated time amounted to approximately 6 h 28 min for manual performance, while the automated process required about 180 min, including the usage of 48 tips and four centrifugation runs (20–35 min each). The one-step approach of PSP loading and labeling was completed by the pipetting robot in about 259 min, including the usage of 61 tips and eight centrifugation runs. In addition to time saving and increased process efficiency, our simple automation approach also enables a high degree of process flexibility, as staining and labeling steps can be performed as two separate workflows or combined into a single continuous workflow using the integrated Bioshaker 3000-T unit.

## Conclusion and outlook

In summary, we established and validated automated workflows for the dye loading and labeling of polymer particles with simple, cost-efficient, and broadly available commercial automation tools such as a self-programmable pipetting robot and a microtiter plate (MTP) reader exemplarily for polystyrene particles (PSP) of different size and surface chemistry and the representatively chosen dyes Nile Red (NR) and 6-aminofluorescein (6-AMF) and fluorescein isothiocyanate (FITC) for core staining and surface labeling. Thereby, we could demonstrate the markedly improved reproducibility, robustness, and reduced operator-related variability achieved by the automation of key work steps compared to the respective manual workflows as well as the considerable time saving and increased process efficiency. In addition, the straightforward integration of loading and labeling steps into a single automated workflow further allowed to reduce the amount of time required for the fabrication of core-stained and surface-labeled beads required for ratiometric analyte sensing. Our simple automation concept, which deliberately relies solely on simple and cost-efficient automation tools available for the broad community of researchers from different disciplines provides a versatile and standardizable platform with minimized operator-induced uncertainties, which can be used for the fabrication of tailor-made particles with increased throughput and better screening options or generally for applications where a strict control over particle recovery and performance of dye loading and labeling is needed. This underlines the considerable potential of this simple automation concept to replace manual workflows in routine particle functionalization.

The presented automated swelling and labeling workflows are not restricted to plain, carboxylated, and aminated PSP. and the underlying well-established conjugation chemistry but can be broadly utilized for organic and inorganic particles of different composition with different surface functionalities. In addition, these workflows can be expanded to the loading of polymer particles with functional nanoparticles such as superparamagnetic iron oxide nanoparticles, semiconductor quantum dots, and lanthanide-based nanoparticles. Moreover, with the integration of meanwhile commercially available hardware tools such as heating elements and shaking units, a completely automated synthesis and surface modification of fluorescent PSP and other particles is feasible, thereby further reducing manual work steps and hands-on time.

We believe that in the future, such simple and affordable ways of integrating automation steps into the fabrication of functional particles using increasingly available and affordable commercial automation tools will pave the road to a more efficient and reproducible production of tailor-made particle systems such as fluorescently stained and labeled beads and ease workflow validation and standardization. Such automated workflows could also facilitate and accelerate the production of particle-based reference materials such as calibration beads for fluorescence microscopy and flow cytometry or model systems for the life and environmental sciences, helping to establish and validate methods for measuring micro- and nanoplastics in complex matrices or assessing life cycles of polymer materials or risk assessment and release studies focused on the environmental fate, transport, and transformation of engineered nano- and advanced materials.

## Supplementary Information

Below is the link to the electronic supplementary material.

Supplementary file1 (DOCX 5.78 MB)

## Data Availability

All data are included in the publication and are also available from the authors by request.
