# Supplementary material for: Validated workflows for preparing and characterizing core-stained and surface-labeled fluorescent polymer particles with simple commercial automation tools
Source: Anal Bioanal Chem. 2026 Mar 18;418(16):5219–31. doi: 10.1007/s00216-026-06443-z (PMC13424375; doi:10.1007/s00216-026-06443-z)
Supplement: Supplementary file 1 — Supplementary file1 (DOCX 5.78 MB) [file 216_2026_6443_MOESM1_ESM.docx]

**Supporting Information**

**Validated Workflows for Preparing and Characterizing Core Stained and Surface Labeled Fluorescent Polymer Particles with Simple Commercial Automation Tools**

Abdelouahad El Abbassi, ^a,b,ǂ^, Paul Fürstenwerth, ^a,b,ǂ^ Christian Würth^a^, Isabella Tavernaro^a,*^, and Ute Resch-Genger^a^,^*^

^a^Division Biophotonics, Federal Institute for Materials Research and Testing (BAM), Richard-Willstätter-Str. 11, 12489 Berlin, Germany.

^b^Department of Biology, Chemistry, and Pharmacy, Free University Berlin, Arnimallee 22, 14195 Berlin, Germany,

^ǂ^The authors contribute equally to this work

*Corresponding authors: Isabella Tavernaro: [isabella.tavernaro@bam.de](mailto:Isabella.tavernaro@bam.de); Ute Resch-Genger: [ute.resch@bam.de](mailto:ute.resch@bam.de)

ORCID ID:

Abdelouahad El Abbassi: 0009-0008-3961-2326

Paul Fürstenwerth: 0009-0002-8656-3279

Christian Würth: 0000-0002-0204-9727

Isabella Tavernaro: 0009-0004-0134-1066

Ute Resch-Genger: 0000-0002-0944-1115

**List of Contents**

[**1.** **Workflow Part 1- Instrumental Setup** 1](#_Toc223448713)

[**1.1 Optimization strategy workflow for the automated liquid handling** 1](#_Toc223448714)

[**1.2 Quality Control Measurements - Microtiterplate Reader** 2](#_Toc223448715)

[**1.2.1 Assessing the Optical Readout Workflow and MTP Performance** 2](#_Toc223448716)

[**2. Determination of Particle Loss** 3](#_Toc223448717)

[**3. Nanoparticles tracking analysis for size determination and validation of the particle number concentration (PNC)** 4](#_Toc223448718)

[**4. Zeta potential analysis of 100 nm PSP before and after NR loading** 6](#_Toc223448719)

[**5. Confocal Laser Scanning Microscopy** 8](#_Toc223448720)

[**6. Determination of the Amount of 6-AMF bound to 6-AMF-Labeled PSP** 9](#_Toc223448721)

[**7. Loading and Labeling of Aminated PSP with NR and FITC** 10](#_Toc223448722)

# **Workflow Part 1- Instrumental Setup**

## **1.1 Optimization strategy workflow for the automated liquid handling**


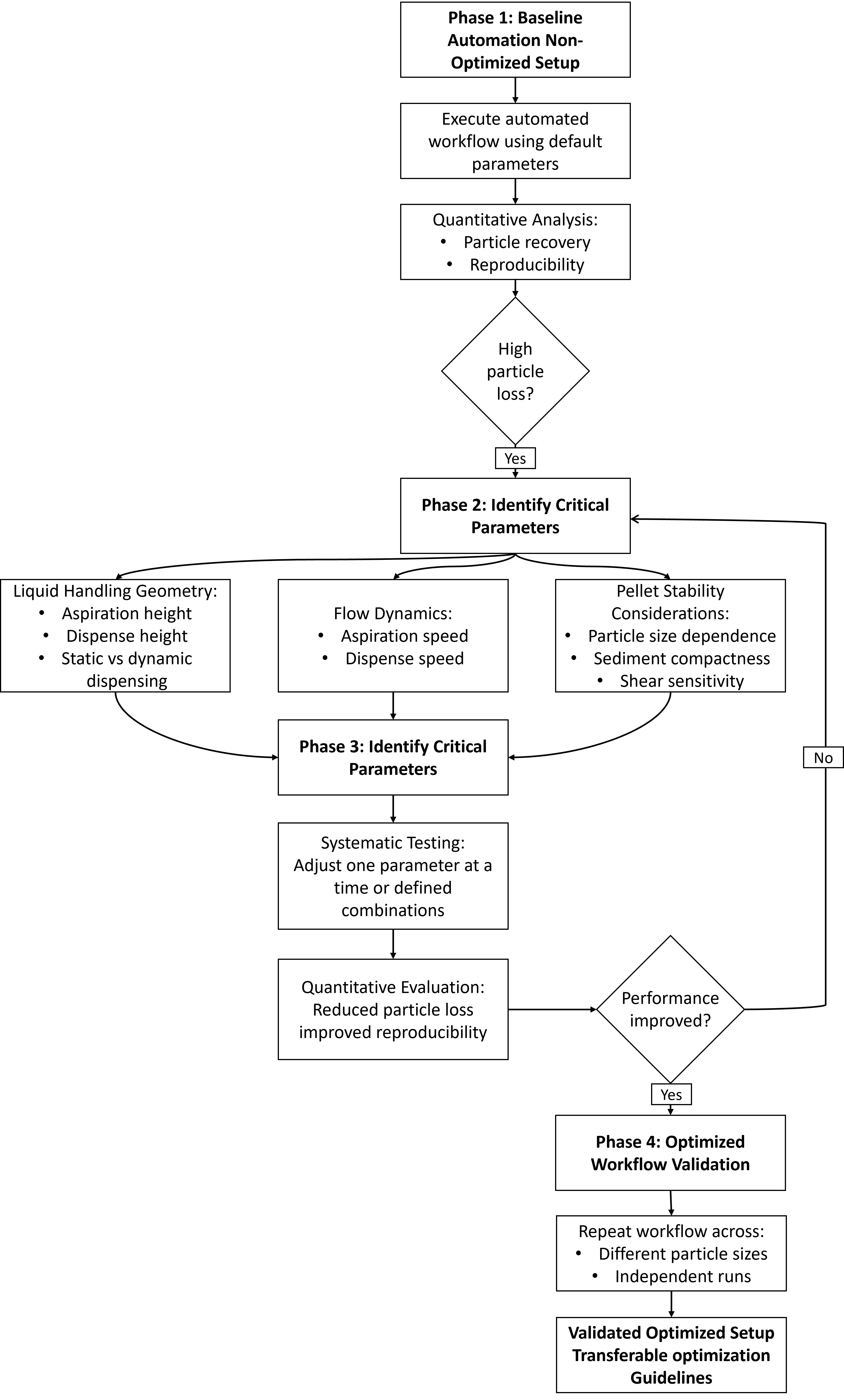


**Figure S1.** Systematic optimization workflow for automated liquid handling of PSP to minimize particles loss and improve process robustness.

## **1.2 Quality Control Measurements - Microtiterplate Reader**

In the first part of the workflow, quality control measurements of the automated setup to reduce potential pipetting errors during transfer and dispense steps were conducted. Therefore, control measurements with a test dye Y, that was validated together with the BAM fluorescence standard kit dyes BAM-F001 to BAM-F005 were performed by the Assist Plus automated pipetting system (INTEGRA Biosciences, Switzerland), equipped with a 1250 µL 8-channel Integra Voyager pipette (accuracy: 125 µL +/- 6.0%; precision: 125 µL; <1.10%) and the specific TipGrips (Integra Biosciences). Following the general rule of adding at least one-third of the maximum fill volume to have an efficient and realistic measurements two different programs were generated with the software VIALAB (version: 3.6.0) for the pipetting robot to transfer and repeatedly dispense different volumes (150 µL, 200 µL, and 250 µL) of blank, standards, and test dye. Depending on the used solvents and needed absorbance/emission wavelength ranges a quartz 96-well microtiter plate (HELMA) or disposable high-binding 96-well microtiter plates (µClear, PS, Chimney Well, Greiner Bio-One, Germany) were used. Readout of absorbance/extinction and fluorescence emission spectra were performed with the Infinite M200 pro microplate reader from Tecan (Switzerland) and the software Tecan i-control (Version 2.0), using absorbance scan and fluorescence intensity scan in “top” mode. Absorbance was measured in a wavelength range from 340-900 nm (steps: 2 nm, flashes: 3), while fluorescence intensity was measured within the standard specifications (steps: 2 nm, flashes read:5; integration time: 20 µs).

### **1.2.1 Assessing the Optical Readout Workflow and MTP Performance**


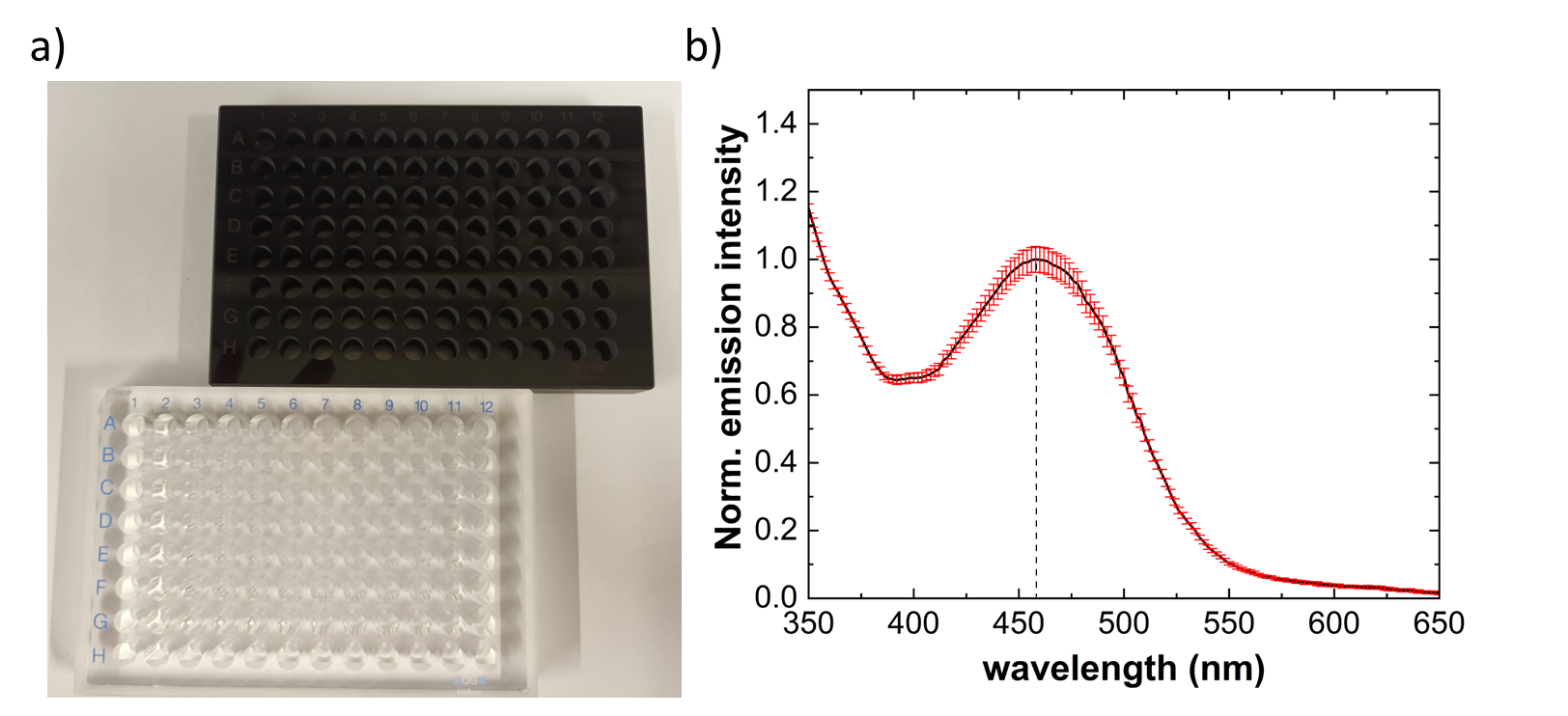


**Figure S1.** (a) Photograph of the transparent and black quartz 96-well microtiter plates used for optical measurements. **(b)** Assessment of the influence of solvent evaporation on absorbance measurements with a 96-well microtiter plate (MTP; quartz, transparent) with the test dye Y dissolved in acetonitrile (ACN). Each well was filled with 200 µL of dye solution (absorbance ~0.04 at the absorption maximum of 468 nm (dashed line), c = 85 µM) immediately after MTP filling revealed matching absorbance spectra and small changes in absorbance intensity

Absorbance measurements performed with a 96 well MTP, with each well being filled with 200 µL of an ACN solution of dye Y with an absorbance ~0.04 at the absorption maximum of 468 nm (c = 85 µM) immediately after MTP filling revealed small well-to-well variations and minor changes in absorbance intensity and relatively closely matching absorbance spectra (Figure S1 b). These measurements, which are representative of the well-to-well variations in absorbance during the measurement of the absorption spectra of each well of a 96 well microtiter plate (MTP), were performed within 12 min.


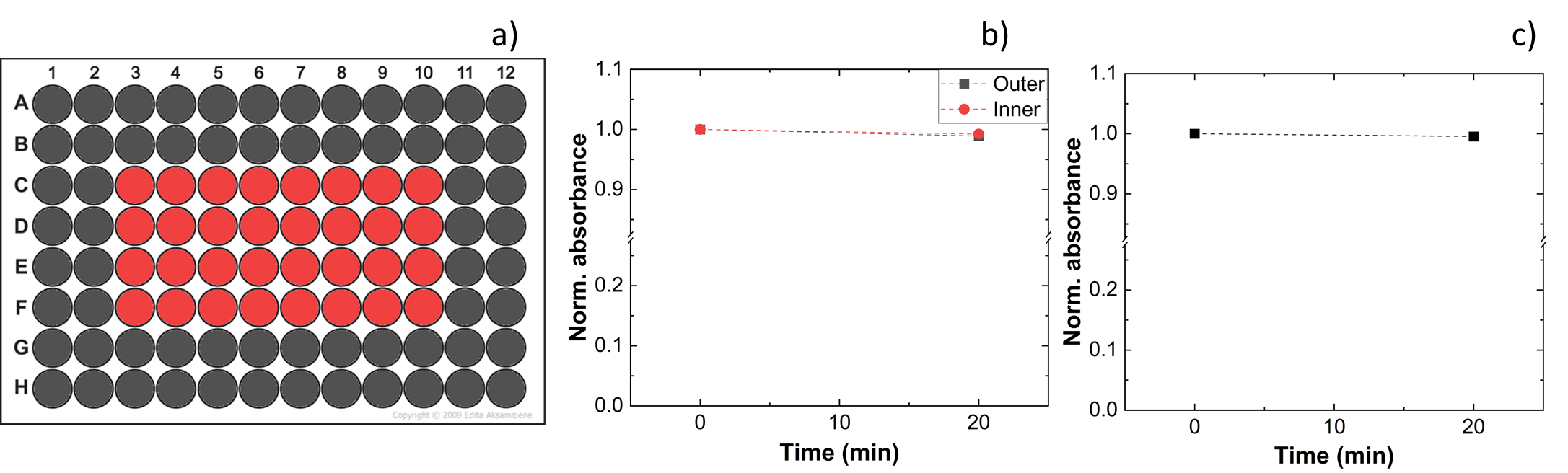


**Figure S2.** Evaluation of the effect of the volatile organic solvent tetrahydrofuran (THF) during measurements with the Tecan Reader M200 MTP reader. a) Sketch of a 96-well plate with differently colored zones representing the outer and inner wells; b) Mean effect of solvent evaporation on the different segments of a 96-well plate over the course of 20 min filled with a 0.001 mM solution of Nile Red (NR) in THF:MilliQ water (4:1; v:v) as derived from the absorption at 532 nm, c) Mean effect of solvent evaporation on the absorption of each well in a 96-well plate over the course of 20 min filled with an ACN dye Y solution (absorbance ~0.04 at the absorption maximum of 468 nm, c = 85 µM) measured with the Tecan Reader M200.

Evaporation of volatile organic solvents is a challenge for performing optical measurements with a microtiter plate as required for the determination of the average dye concentration per particle after dye extraction from the initial dye-loaded PSP done by dissolving an aliquot of the particle samples in THF, resulting in a solvent mixture of THF and MilliQ-water (4:1, v/v). These dye solutions were measured in a 96-well quartz microtiter plates (see Figure S1 a)) to enable faster throughput due to the large number of samples. During the loading and preparation of the microtiter plate, the volatile components could evaporate, altering the dye concentration inside the wells and the filling height (optical pathlength). As shown in Figure S2(b) and (c), the measured absorbance values decreased over time, with the corresponding small changes amounting to about 1% over the observed time period of 20 min for both dyes in the chosen solvent systems. While these findings barely affect the results obtained with the Tecan Reader M200, they need to be considered in the calculation of the measurement uncertainties.


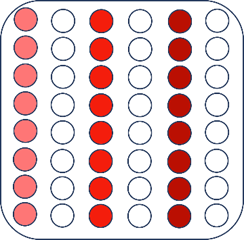


**Figure S3**. Determination of the Standard Deviation (SD) of the MTP measurements performed to determine dye loading concentrations. Representative sketch of the Eppendorf tube rack used in the swelling experiments. Each color corresponds to a different Nile Red (NR) concentration, with the increasing intensity of the red color indicating higher concentrations. For each NR concentration, eight independent samples were prepared to assess the reproducibility of the measurements.

The standard deviation (SD) reflects the variation between the 8 replicate samples in a row (Figure S3).

# **2. Determination of Particle Loss**

The gravimetric measurements were performed in duplicate. In total, 16 dried samples were obtained and weighed for each dye concentration (variation 1) and each size (variation 2). The SD values reported for the gravimetric study therefore reflect the variation in recovered bead mass across these dried replicates.

The determined particle mass concentrations for NR-loaded, 100 nm and 1000 nm sized PSP were averaged over 3 separate runs and did not significantly change. For the 100 nm PSP, the average mass concentration is 1.5 ± 0.04 mg and for the 1000 nm samples, the average mass concentration is 2.4 ± 0.03 mg, see Table S1.

**Table S1.**Gravimetric determination of polystyrene particle mass after swelling for 100 nm and 1000 nm PSP: mean ± SD of eight replicates per dye concentration for three independent runs.


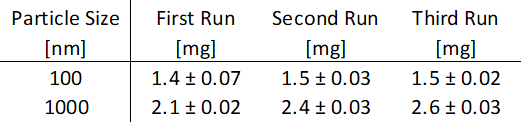


# **3. Nanoparticles tracking analysis for size determination and validation of the particle number concentration (PNC)**

Particle size and particle number concentration (PNC) were determined by nanoparticle tracking analysis (NTA). In comparison to other techniques like dynamic light scattering (DLS), NTA offers superior resolution in size distribution profiling, enabling detailed characterization of heterogeneous nanoparticle populations. A key advantage of NTA is its ability to measure fluorescently labeled particles, allowing selective detection in complex or multi-component systems. However, NTA is generally more time-consuming, requires careful sample dilution to maintain optimal particle visibility, and is limited by a narrower size range and lower throughput compared to DLS. Measurements were conducted with the NanoSight LM 10 system from Malvern Panalytical (Germany) equipped with a 405 nm laser and the software NanoSight Version 3.2 at a temperature of 25 °C in static mode, following the standards ISO19430 and ASTM E2834.(1, 2). The NanoSight NTA software (Version: 3.32) was used to capture 5 videos with 60 s and 25 fps of the scattering of the highly diluted samples.

Prior samples measurements, performance qualification (PQ) measurements with a particle size standard (polystyrene particles PS-ST-B1261) from microparticles GmbH (Germany) were performed to confirm validation of the setup (Figure S4).


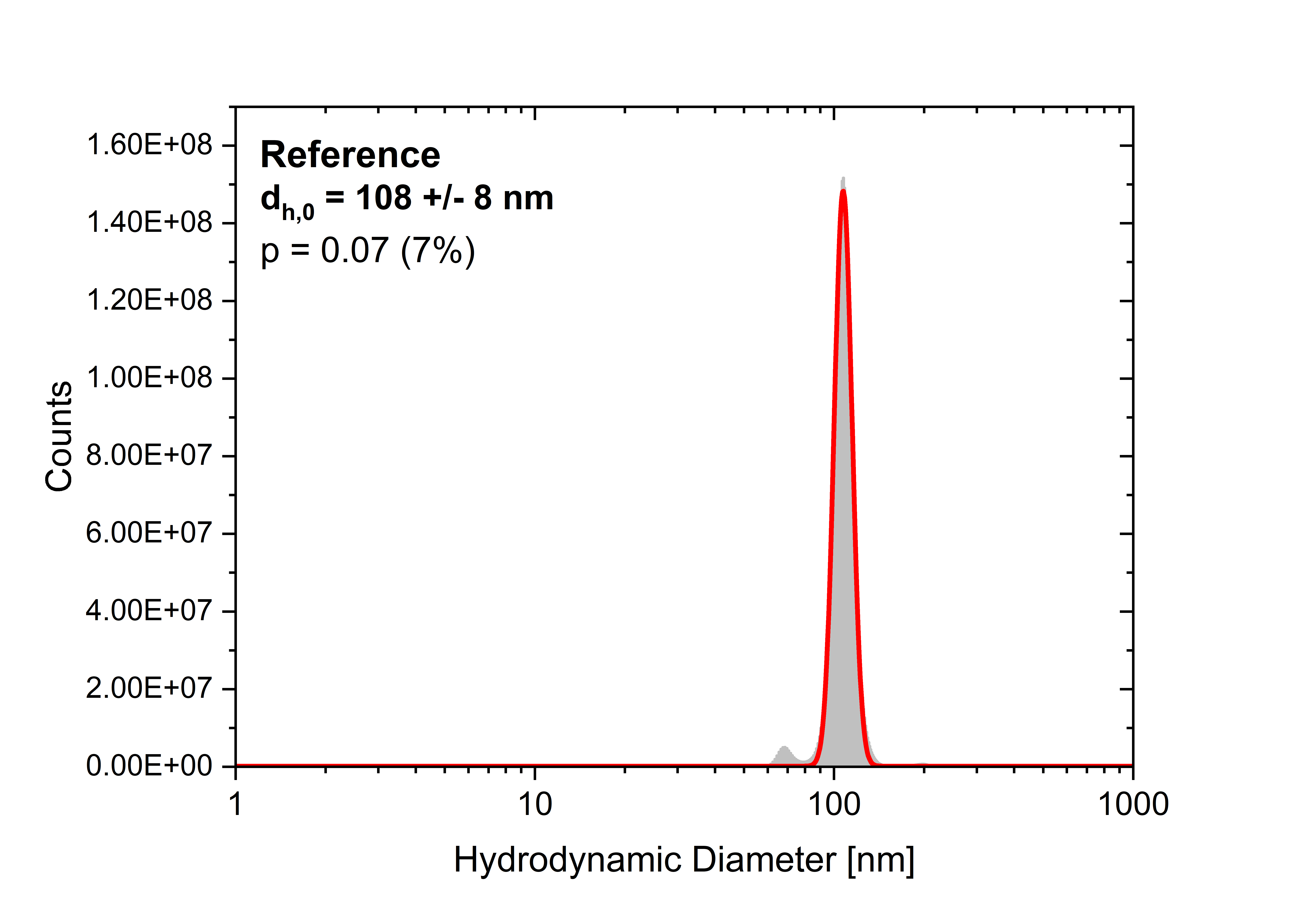


**Figure S4.** Results of the PQ measurements with a particle size standard.

After the PNC was gravimetrically determined by measuring the total particle mass and converting it to particle number based on the assumed particle size and density, this approach was validated by NTA measurements. The gravimetric results were compared with the NTA data to identify potential deviations caused by particle aggregation, incorrect density assumptions or incomplete dispersion. This comparison, shown in Figure S5 ensured the reliability of the gravimetric method and confirmed its consistency with particle-resolved analytical measurements.


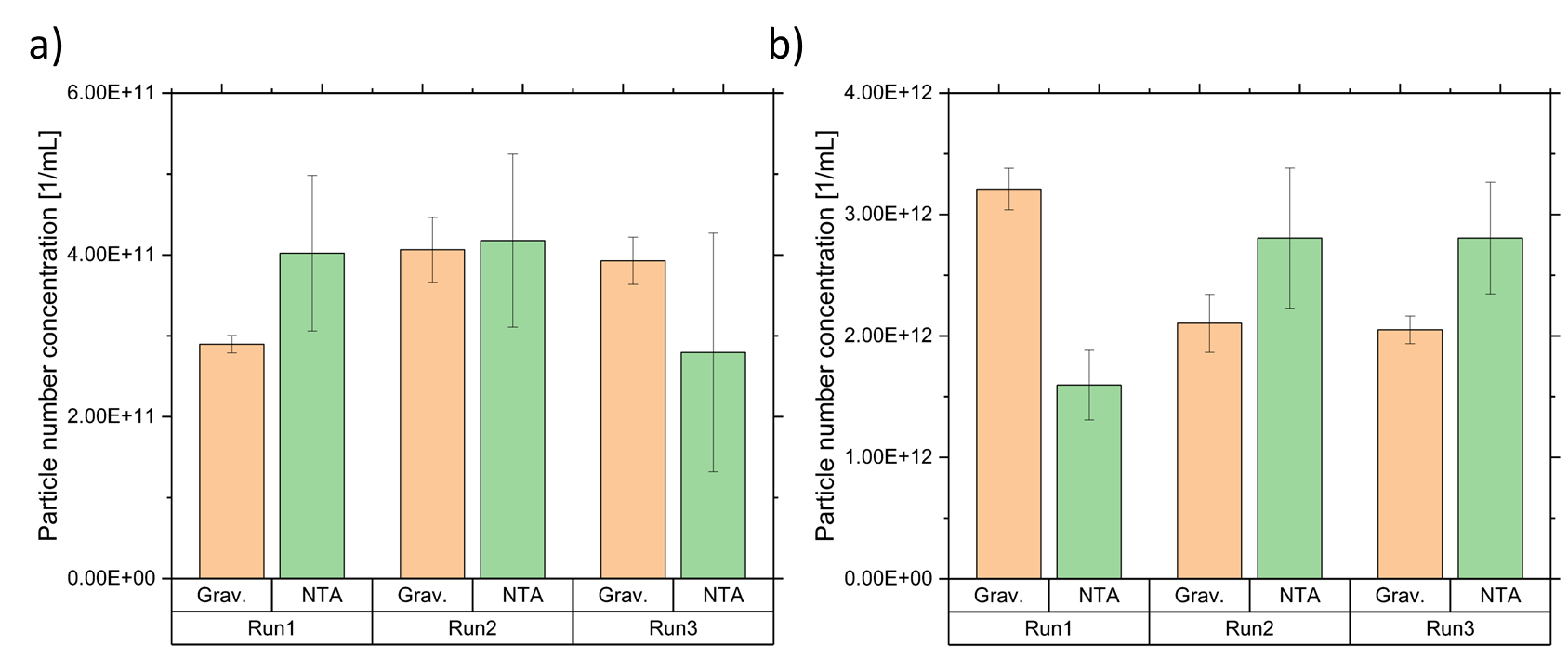


**Figure S5.** Comparison of the PNC determined by NTA and gravimetrically (Grav.) for a) 200 nm PSPs and b) 100 nm PSP. b) For each individual run. The bars represent mean ± SD from three independent runs (n = 24 per run).

To study potential influences of the automated swelling approach, the number-based hydrodynamic diameters (d_h,0_) of the PSP were measured by NTA and compared with the neat particles (Figure S6). Only aqueous dispersions of the 100 nm and 200 nm sized NR-loaded PSP were characterized by NTA.


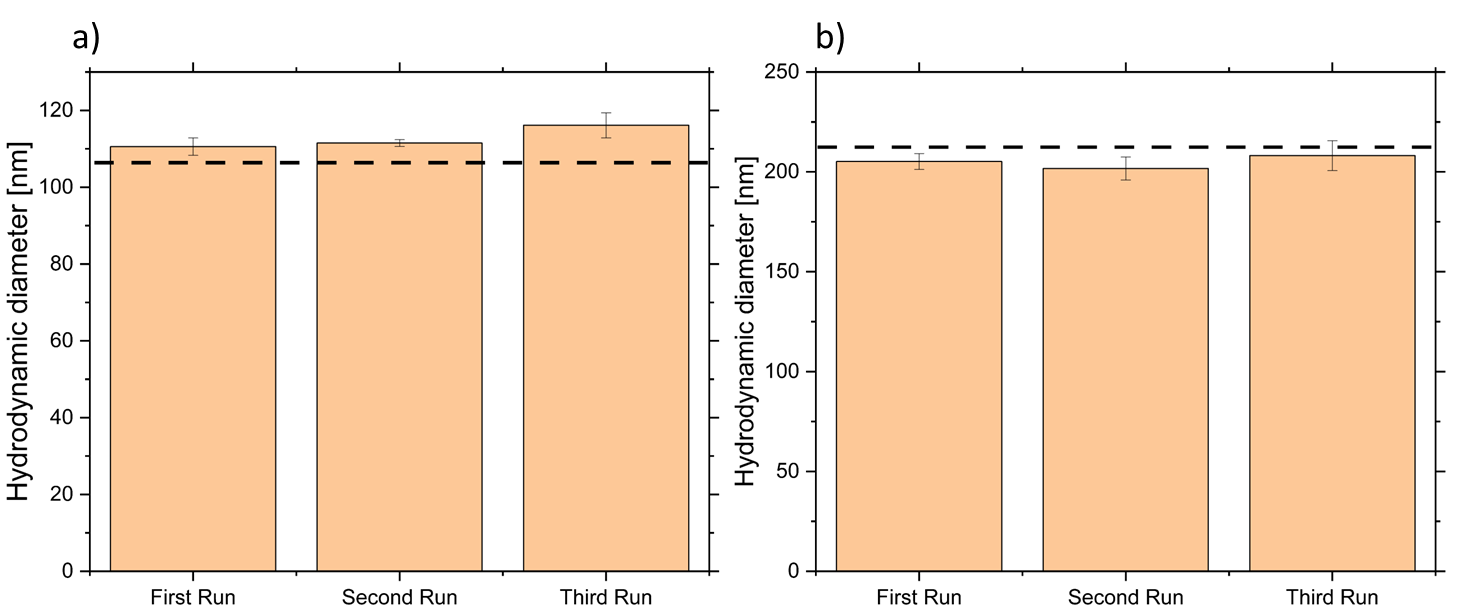


**Figure S6.** Comparison of the number-based hydrodynamic diameter (d_h,0_) determined via NTA measurements for the subsequent runs of the a) 200 nm and b) 100 nm NR-loaded PSP with the dashed line representing the determined hydrodynamic diameter for the neat particles. The bars represent mean ± SD from three independent runs (n = 24 per run).

# **4. Zeta potential analysis of 100 nm PSP before and after NR loading**

Zeta potential measurements were performed for 100 nm PSP before and after the swelling and NR loading procedure, to evaluate a possible adsorption of NR to the PSP surface for the resulting dye-loaded PSP. The obtained values remained unchanged within the experimental uncertainty across all samples assessed, supporting that the swelling procedure does not significantly alter the surface charge of the particles. This confirms the assumption that no significant amount of NR is adsorbed onto the PSP surface.


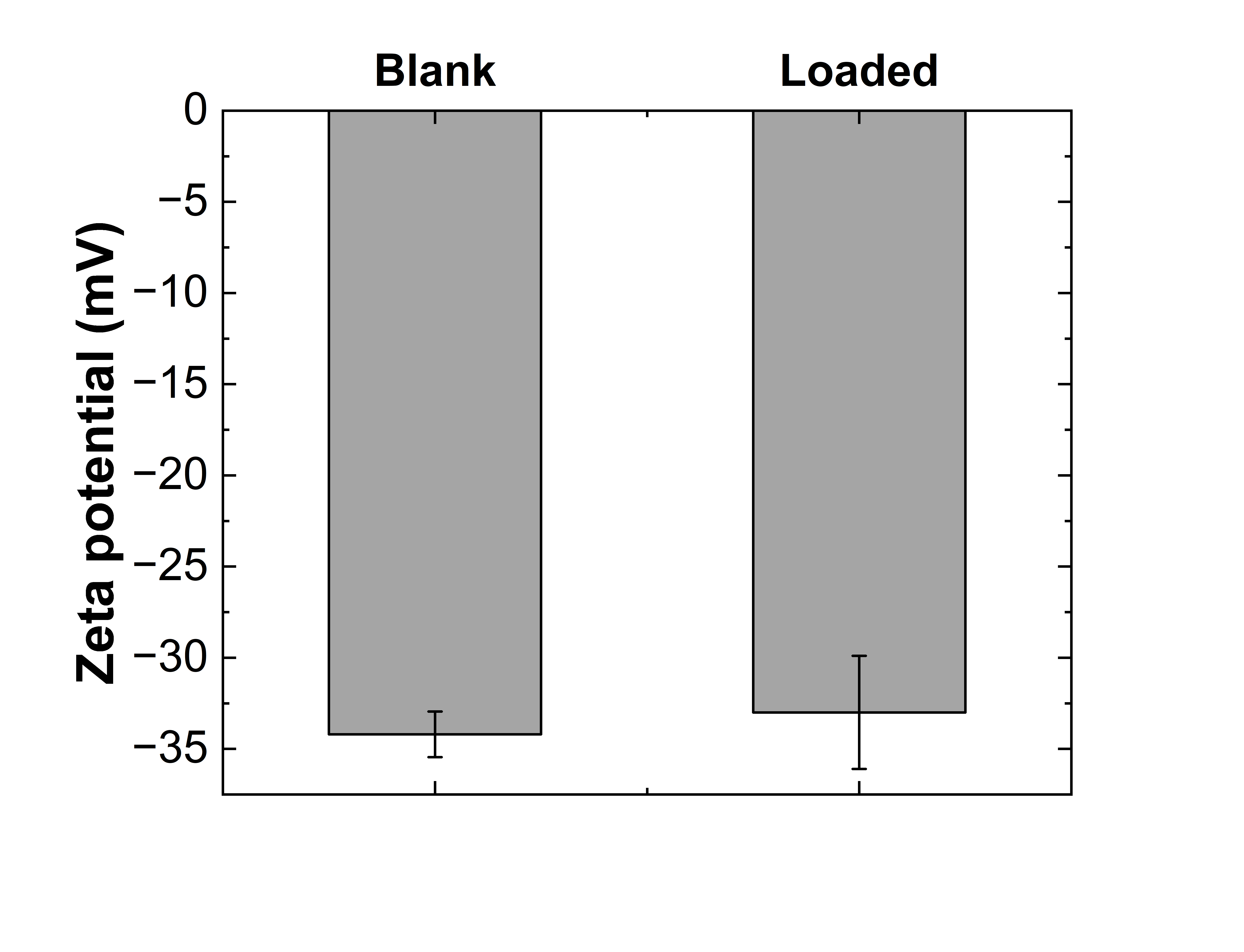


**Figure S8.** Zeta potential of 100 nm before (blank) and after NR loading at different initial dye concentrations. The values represent mean ± SD (n = 24).

# **5. Confocal Laser Scanning Microscopy**

Confocal laser scanning microscopy (CLSM) was employed to qualitatively assess dye-loading and -labeling of the PSP core stained with hydrophobic NR and surface labelled with hydrophilic 6-AMF. Imaging was performed using an Olympus FV-3000 confocal microscope equipped with a 100 xoil immersion objective, providing high-resolution optical sectioning and fluorescence detection. CLSM enabled precise localization of NR (λ_ex_ = 561 nm, λ_ex_ = 570-670 nm) in the bead core and 6-AMF near and on the surface (λ_ex_ = 488 nm, λ_em_ = 520 -550 nm), confirming successful incorporation and spatial separation of dyes (Figure S7). This qualitative approach serves as a rapid and reliable method for verifying dye-loading uniformity prior sensing applications.


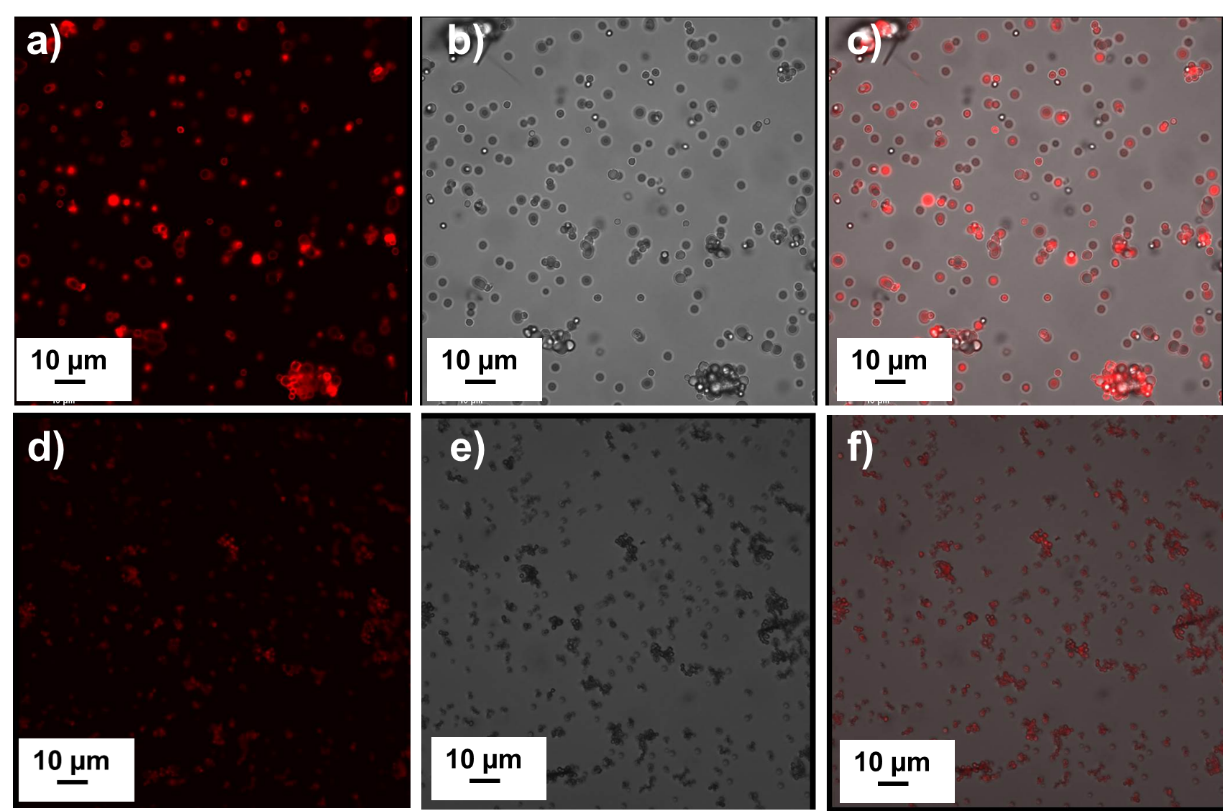


**Figure S9.** CLSM images of NR-loaded carboxylated PSP (1 µm; a-c) and aminated PSP (1 µm, d-f).


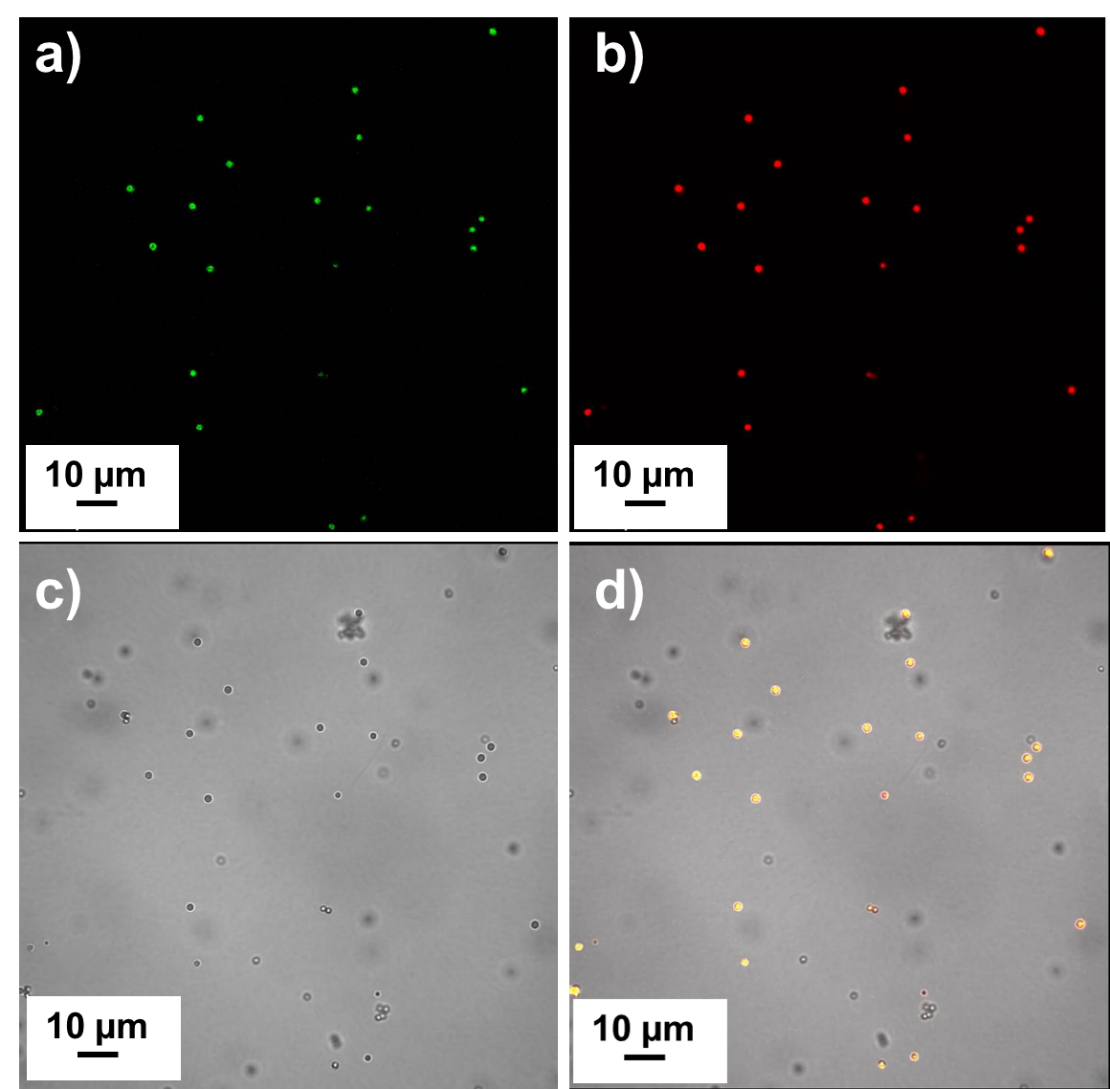


**Figure S10.** CLSM images of NR-loaded and 6-AMF-labeled PSP (1 µm).

# **6. Determination of the Amount of 6-AMF bound to 6-AMF-Labeled PSP**

To determine the amount of 6-AMF covalently attached to the carboxylic acid groups at the PSP surface of carboxylated PSP, the measured absorption of the dissolved PSPs in MeOH was compared with a calibration curve shown in Figure S9 a). After consideration of the dilution factor, the amount of 6-AMF was determined for five different particle batches. Figure S9 b). The labeling amount of 6-AMF is consistent across the 5 measured samples, spreading around the mean amount of 31 nmol, leading to a standard deviation of ± 4 nmol**.** This supports the reproducibility and robustness of the developed automatic labeling method with 6-AMF.


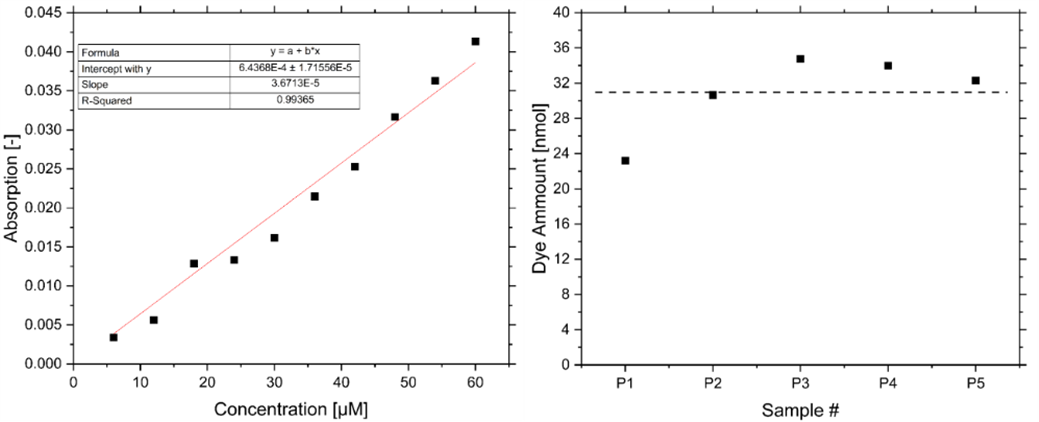


**Figure S11.** Left: Calibration curve used for the determination of 6-AMF in MeOH attached to the surface of the NR stained carboxylated PSP. The absorbance was recorded at 480 nm. Right: Variations in the amount of 6-AMF in each sample around the mean amount of 31 nmol (dashed line) determined as average value from these measurements.

# **7. Loading and Labeling of Aminated PSP with NR and FITC**

Labeling the particle surface is a versatile and adaptable procedure, which can be implemented for a variety of different systems. Such a system can be an aminated particle, in which the particle surface is functionalized with primary amino groups, leading to a positively charged particle surface.

To covalently bind a dye with an orthogonal functionality or reactive group dye to particle surface functionalities, a similar procedure as described in Section 5. can be used. For the covalent attachment to amino groups on PSP, a different pH-responsive fluorescein derivative was used, fluorescein isothiocyanate (FITC) with a reactive isothiocyanate group instead of an amino group as utilized for the labeling of carboxylated PSP. First, the aminated particles were loaded in an identical way as done for the carboxylated PSP with different concentrations of NR, using 0.1 µM, 1 µM, 10 µM, and 100 µM, NR and 8 samples for each concentration. Second, the labeling procedure was performed by adding the NR-loaded, aminated particles to a reaction mixture containing different concentrations of FITC of 0.15 mM and 0.30 mM. This leads to the formation of a thioamide bond between the fluorescein dye and the PSP surface groups, thereby covalently binding FITC to the particle surface.

Optical measurements using aminated PSPs with a size of 1000 nm were used stained with varying concentrations of NR and FITC, leading to a set of differently emissive particle systems shown in Figure S 10. The NR and FITC emission peak at 512 nm and 562 nm, respectively, similar to the carboxylated PSPs shown in Section 5 and in Figure 6 in the main manuscript. This highlights the variability of the used and implemented method, which provides a versatile platform to efficiently produce a large variety of automatically stained and labeled particle systems of varying size, surface chemistry, emission color, and sensing features.


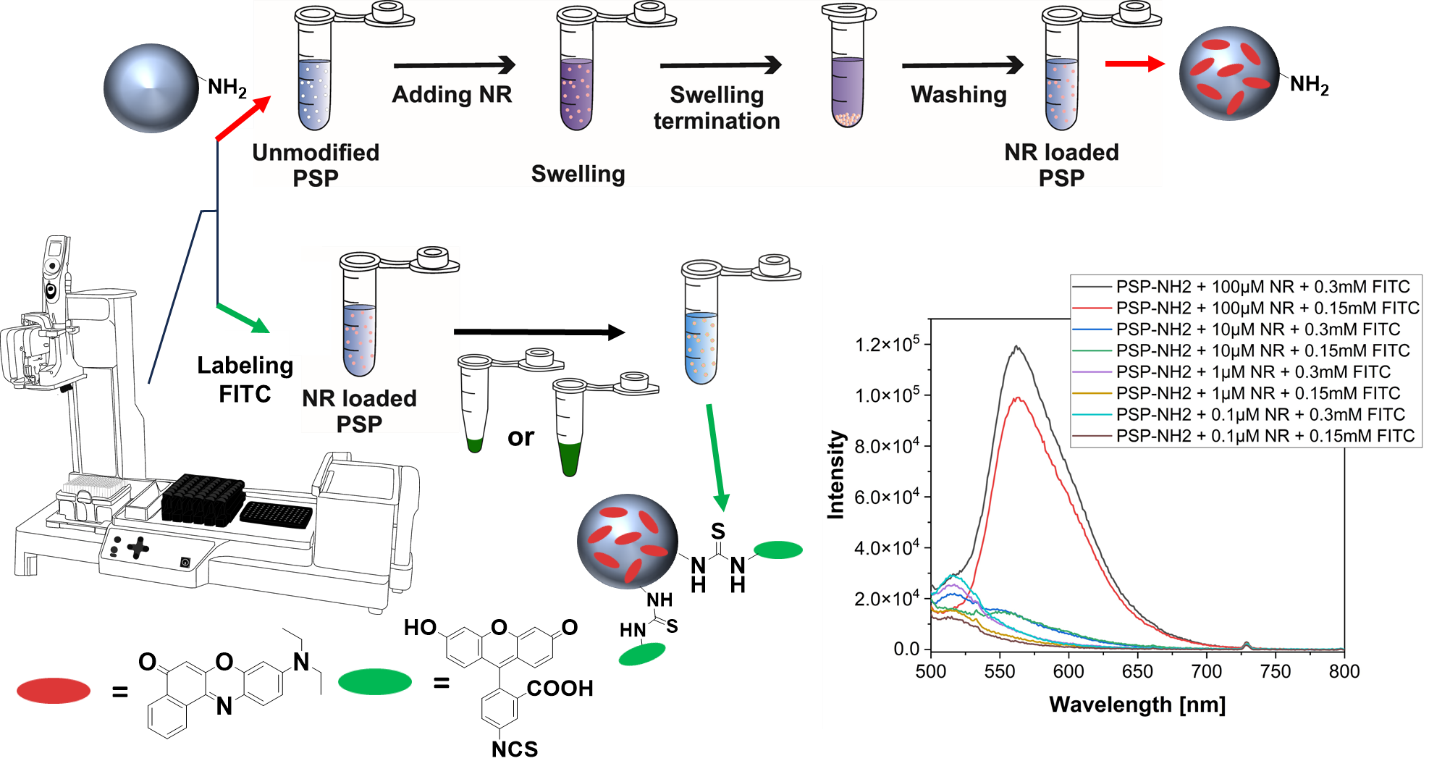


**Figure S12.** Overview of the workflow used for the NR loading (NR) and FITC labeling of aminated polystyrene particles. The emission spectra recorded upon excitation at 480 nm reveal the different intensities resulting from the different staining and labeling concentrations.

**References**

1. International A. ASTM E2834-12 — Standard Guide for Measurement of Particle Size Distribution of Nanomaterials in Suspension by Nanoparticle Tracking Analysis (NTA). Original (2012 version) ed. West Conshohocken, PA, USA: ASTM International; 2012.

2. (ISO) IOfS. ISO 19430:2016 — Particle size analysis — Particle tracking analysis (PTA) method. 1 ed. Geneva, Switzerland: ISO; 2016.
